# Discovery and evaluation of inhibitor of LARP6 as specific antifibrotic compound

**DOI:** 10.1038/s41598-018-36841-y

**Published:** 2019-01-23

**Authors:** Branko Stefanovic, Zarko Manojlovic, Cynthia Vied, Crystal-Dawn Badger, Lela Stefanovic

**Affiliations:** 1 Department of Biomedical Sciences, College of Medicine, Florida State University, 1115 West Call Street, Tallahassee, FL 32306 USA; 2 Present Address: Keck School of Medicine of University of Southern California, 1450 Biggy Street, NRT 4510, Los Angeles, CA 90033 USA; 3 Translational Science Laboratory, College of Medicine, Florida State University, 1115 West Call Street, Tallahassee, FL 32306 USA; 4 Present Address: Proteomics and Metabolomics Facility, Colorado State University, 401 West Pitkin Street, Fort Collins, CO 80521 USA

## Abstract

Fibrosis is characterized by excessive production of type I collagen. Biosynthesis of type I collagen in fibrosis is augmented by binding of protein LARP6 to the 5′ stem-loop structure (5′SL), which is found exclusively in type I collagen mRNAs. A high throughput screen was performed to discover inhibitors of LARP6 binding to 5′SL, as potential antifibrotic drugs. The screen yielded one compound (C9) which was able to dissociate LARP6 from 5′ SL RNA *in vitro* and to inactivate the binding of endogenous LARP6 in cells. Treatment of hepatic stellate cells (liver cells responsible for fibrosis) with nM concentrations of C9 reduced secretion of type I collagen. In precision cut liver slices, as an *ex vivo* model of hepatic fibrosis, C9 attenuated the profibrotic response at 1 μM. In prophylactic and therapeutic animal models of hepatic fibrosis C9 prevented development of fibrosis or hindered the progression of ongoing fibrosis when administered at 1 mg/kg. Toxicogenetics analysis revealed that only 42 liver genes changed expression after administration of C9 for 4 weeks, suggesting minimal off target effects. Based on these results, C9 represents the first LARP6 inhibitor with significant antifibrotic activity.

## Introduction

Fibrosis is characterized by excessive synthesis of type I collagen in various organs and major complications of fibrosis are direct result of massive deposition of type I collagen in the extracellular matrix^1,2^. The disease is progressive, but currently there is no therapeutic approach to directly and specifically inhibit excessive synthesis of type I collagen. Reversal of fibrosis is only possible at early stages when the crosslinking between collagen fibers is still in immature state^3,4^.

The goal of optimal antifibrotic therapy is to inhibit type I collagen production only in fibrotic lesions and spare the constitutive type I collagen synthesis. However, current approaches mostly target the pleiotropic TGFβ, CTGF, PDGF, Wnt, or Notch signaling pathways^5–9^ or are based on antibody mediated inhibition of lysyl oxidase-like 2 (LOXL2) enzyme^10,11^ As antifibrotic therapy must be applied for prolonged periods of time, antifibrotic drugs must have minimal side effects, must specifically target excessive type I collagen synthesis and must be affordable; the requirements which current approaches are lacking.

Type I collagen is a heterotrimer composed of two α1(I) and one α2(I) polypeptides and is one of the most stable proteins in human body with half-life of 4–12 months. Its fractional synthesis rate (defined as % synthesis per day) is about 2% in the skin^12^, while in the liver it is only 0.2%^13^. This low grade, constitutive, synthesis is in contrast with synthesis in fibrosis, where type I collagen production can be increased several hundred fold^14,15^. The dramatically increased rate of type I collagen synthesis in fibrosis is not merely an augmentation of the constitutive synthesis; an additional mechanism must be activated^15–22^. The key molecular interaction activating this mechanisms is binding of protein LARP6 to the mRNAs encoding type I collagen^23^. Collagen α1(I) mRNA and α2(I) mRNA have an evolutionary conserved secondary structure in their 5′ UTR, the 5′ stem-loop (5′SL). 5′SL is not found in any other mRNA, only type III collagen mRNA has a similar structure^24^. 5′SL binds RNA binding protein LARP6 with high affinity and with strict sequence specificity^23,25,26^. 5′SL is the only known target of LARP6, which serves as an adapter protein that recruits accessory translational factors to increase translational competency of type I collagen mRNAs and to couple translation of collagen α1(I) polypeptide to that of α2(I) polypeptide^17–21,23,25,27–29^. The coupled translation of collagen α1(I) and α2(I) mRNA results in production of collagen polypeptides at discrete sites on the endoplasmic reticulum (ER) membrane. This facilitates their folding into type I collagen, resulting in rapid excretion of the protein into the extracellular matrix.

The importance of LARP6 dependent regulation of type I collagen in hepatic fibrosis came from creation of the 5′SL knock in mice^30^. In these animals a mutation was introduced into collagen α1(I) gene which changed the nucleotides encoding the 5′SL. The mutation did not change the coding region of the gene nor the expression level of the mRNA. Thus, in the homozygous knock in mice synthesis of collagen α1(I) polypeptide is not subjected to the LARP6 dependent regulation. The 5′SL knock in mice develop normally and have no abnormalities, proving that constitutive collagen synthesis is not compromised. However, these animals are resistant to development of hepatic fibrosis; hepatic fibrosis induced by bile duct ligation in these animals was greatly reduced compared to the wt littermates^30^. Hepatic stellate cells (HSCs) are liver cells responsible for type I collagen synthesis in hepatic fibrosis. HSCs from 5′SL knock in mice are able to produce only low amounts of type I collagen, explaining the mild fibrosis^30^. These findings supported the concept that biosynthesis of type I collagen in fibrosis requires binding of LARP6 to collagen mRNAs and bolstered the efforts to find inhibitors of LARP6 binding as specific antifibrotic drugs^31^.

Here we present the discovery, characterization and antifibrotic activity of a chemical compound which was identified in a screen for inhibitors of LARP6 binding to 5′SL RNA.

## Results

### High throughput screen for inhibitors of LARP6 binding

To discover inhibitors of LARP6 binding to 5′SL of type I collagen mRNAs we used *in vitro* high throughput assay based on fluorescence polarization (FP)^32^. When recombinant LARP6 containing the minimal sequence sufficient to bind 5′SL (amino-acids 72–303 of LARP6, here designated LARP6*) is added to fluorescently labeled synthetic 5′SL RNA (fl-5′SL RNA) there is an increase in FP of 100–150 mFP, what is a readout of LARP6 binding. When LARP6* is competed out by unlabeled 5′SL RNA, the FP decreases, indicating the dissociation of the protein from fl-5′SL RNA. Therefore, by measuring a decrease of FP, inhibitors of LARP6 binding to 5′SL RNA could be discovered^32^. Using this assay we screened ChemBridge Inc., DIVERSet-CL chemical library, code NT1174, containing 50,000 drug like compounds. The compounds were added at 200 μM to the 384-well plates containing LARP6* bound to fl-5SL RNA and FP was measured. The average FP (screen FP) and standard deviation (SD) was calculated and a decrease of >3 SD from the screen FP was used as a cutoff value indicating LARP6 dissociation. By using this criterion, after the initial screen of 50,000 compounds, 327 compounds were scored as hits (0.65%). Supplementary Figure 1 shows the mean FP of controls without compounds, the mean of all FP readings of the screen (screen FP) and the cut off limit of the screen (screen FP – 3 SD), together with the readings obtained for C9, C10 and C11 compounds. The FP reading of the controls without compounds and the screen FP were similar, except the SD for screen FP was larger (Supplemental Fig. 1), therefore we used the later to set the more stringent cutoff value.

**Figure 1:**
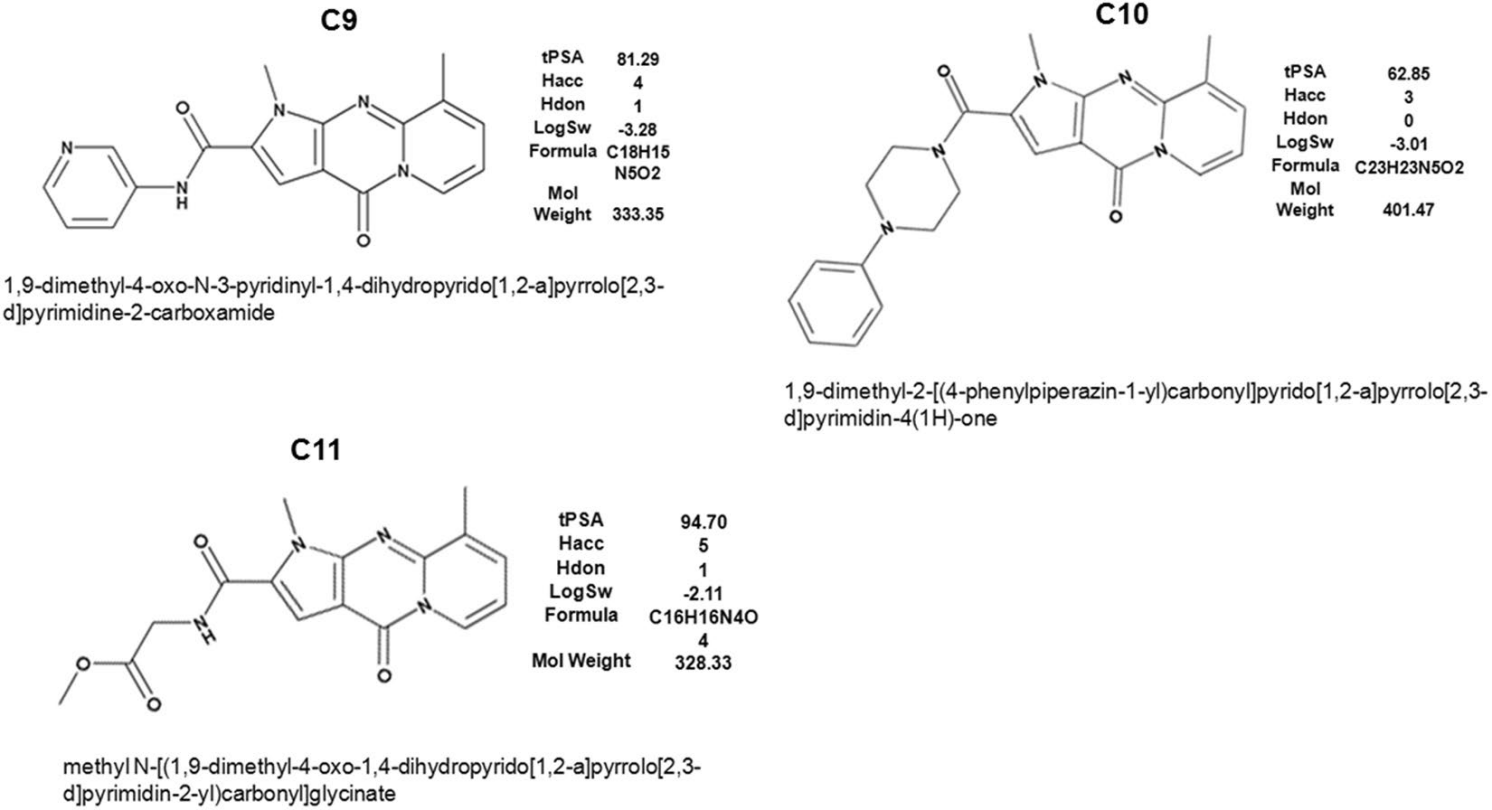
Structure of chemical compounds identified in high throughput screen. The structure of three compounds with a common tri-cyclic core. Their chemical names and drug-like parameters (tPSA, topological polar surface area; Hacc, number of H-bond acceptors; Hdon, number of H-bond donors; LogSw, logarithm of solubility in water, mol/L) are indicated.

The hits were re-screened at 20 μM concentrations and 70 compounds passed this test. Three of these compounds (C9, C10 and C11) had a similar core tri-cyclic structure (Fig. 1), suggesting that the screen has selected for the structurally related compounds. These three compounds were tested for their effect on type I collagen production by human lung fibroblasts (HLFs)^33^ and only one, the compound C9, has reproducibly shown an inhibitory effect. The rest of the results in this manuscript describe the activity of C9 *in vitro* and *in vivo*.

### C9 inhibits binding of LARP6* to 5′SL RNA *in vitro*

**Figure 2:**
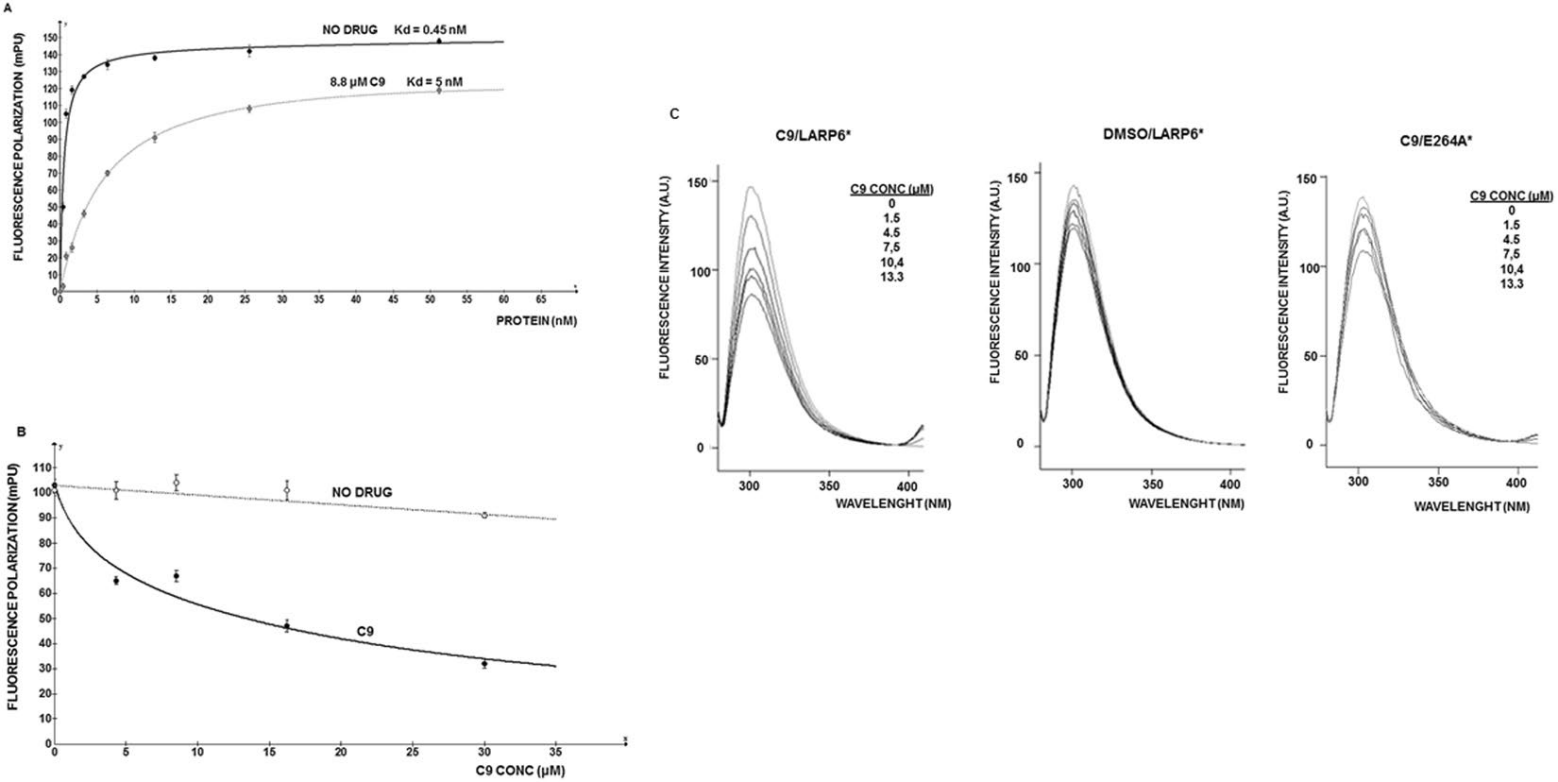
Effect of C9 on binding of LARP6* to 5′SL RNA measured by fluorescence polarization (FP). (**A**) Inhibition of LARP6* binding to 5′SL RNA. Saturation of 1 nM fluorescently labeled 5′SL RNA (fl-5′SL RNA) with increasing amounts of recombinant LARP6* in the absence (full line) or after preincubation with 8.8 μM of C9 (dotted line). (**B**) Dissociation of LARP6* bound to fl-5′SL RNA. LARP6* was bound to fl-5′SL RNA at the saturating concentration and increasing amounts of C9 were added to the reaction and FP measured (full line). Dotted line; dissociation of LARP6* in the absence of drug. (**C**) Change in intrinsic fluorescence of LARP6* tryptophans with increasing concentrations of C9. Left panel: increasing amounts of C9 were added to a cuvette containing wt LARP6* and the fluorescence spectrum of tryptophans was recorded after each addition of C9 (A.U., arbitrary units). The final concentrations of C9 after each addition are indicated. The Stern/Volmer plot is shown in the bottom panel. Middle panel: titration of vehicle (DMSO) into the wt LARP6*. Right panel: titration of C9 into PBS.

The FP readings were used to assess the dose response of C9 on LARP6* binding. Figure 2A, full line, shows a saturation curve of LARP6* binding to fl-5′SL RNA. In the absence of C9 the apparent Kd of 0.45 nM was estimated. Such high affinity of LARP6* binding to 5′SL RNA has been reported before^25^. When C9 was added at 8.8 µM into the binding reaction containing LARP6*, followed by the addition of fl-5′SL RNA, the affinity of LARP6* for 5′SL RNA was reduced ~10 fold (Fig. 2A, dotted line). When LARP6* was first bound to 5′SL RNA at the saturating concentration and C9 was added in increasing concentrations to the preformed complex, the dissociation of LARP6* was observed with IC_50_ of ~15 µM (Fig. 2B). These results suggested that C9 can prevent binding of LARP6* to 5′SL RNA. In the original high throughput screen the compounds were tested at 200 µM, the concentration which is ~13 fold higher than the IC_50_ of C9.

In order to reduce binding affinity of LARP6* C9 may interact with LARP6*, with 5′SL RNA or with both. We could not find any evidence that C9 interacts with 5′SL RNA, however, when C9 was added in increasing concentrations to the recombinant LARP6* a change in the intrinsic fluorescence of LARP6* tryptophans was observed. Figure 2C, left panel, shows quenching of the intrinsic fluorescence of LARP6* tryptophans after progressive addition of C9 to the protein. The Stern/Volmer plot^34^ showed a linear relationship between the fluorescence intensity and C9 concentration (Supplemental Fig. 2A), suggesting the absence of inner filter effect. Middle panel shows titration of vehicle and right panel shows titration of C9 into the LARP6* mutant E264A. This mutant has decreased binding affinity to 5′SL^25^ and its tryptophans are affected to a lesser extent by addition of C9. Titration of C9 into buffer is shown in Supplemental Fig. 2B. To assess the selectivity of C9 over other members of LARP superfamily, we titrated C9 into LARP4 protein. Only minimal change in tryptophan fluorescence of LARP4 was observed (Supplemental Figs 2C and D). Overall, these results suggest that C9 may act to alter conformation of LARP6.

### Binding of endogenous LARP6 is diminished by treatment of cells with C9

**Figure 3:**
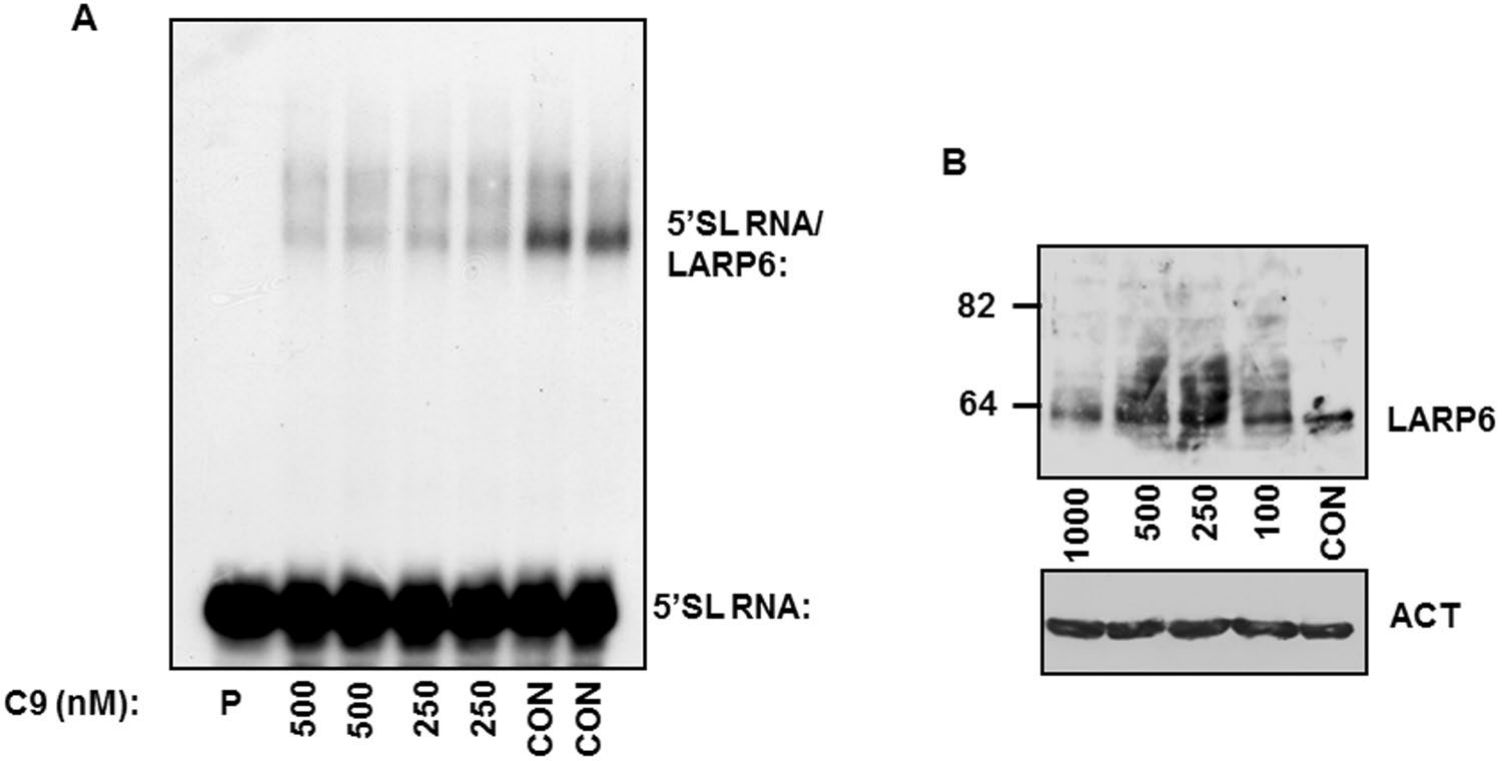
Inhibition of binding of the endogenous LARP6 in cell extracts. (**A**) Gel mobility shift experiment with extracts of human lung fibroblasts (HLFs) treated with the indicated concentrations of C9 and using radiolabeled 5′SL RNA as probe. The migration of free RNA and 5′SL RNA/LARP6 complex is indicated. (**B**) Altered electrophoretic mobility of endogenous LARP6 after C9 treatment of cells. Western blot of LARP6 in the extracts from A. Migration of LARP6 is indicated.

To assess if C9 can inactivate 5′SL binding activity of LARP6 in cells we treated human lung fibroblasts (HLFs) with 250 nM and 500 nM of C9 and analyzed the binding of endogenous LARP6 in cell extracts by gel mobility shift experiments (Fig. 3A)^25^. In extracts of untreated cells binding of LARP6 to radiolabeled 5′SL RNA was clearly detectable (Fig. 3A, CON lanes), however, pretreatment of cells with 250 nM or 500 nM of C9 greatly reduced the ability of endogenous LARP6 to bind 5′SL RNA. When we measured LARP6 protein in the extracts of cells treated with C9 by western blot, the total amount of LARP6 was not decreased, but LARP6 isoforms with higher molecular weight were also detected (Fig. 3B). Preliminary results suggested that these species represent ubiquitinated protein. Overall, these results indicated that C9 is effective in inhibiting LARP6 binding to 5′SL RNA when added at nano-M concentrations to the HLFs.

### C9 inhibits type I collagen production by hepatic stellate cells (HSCs)

**Figure 4:**
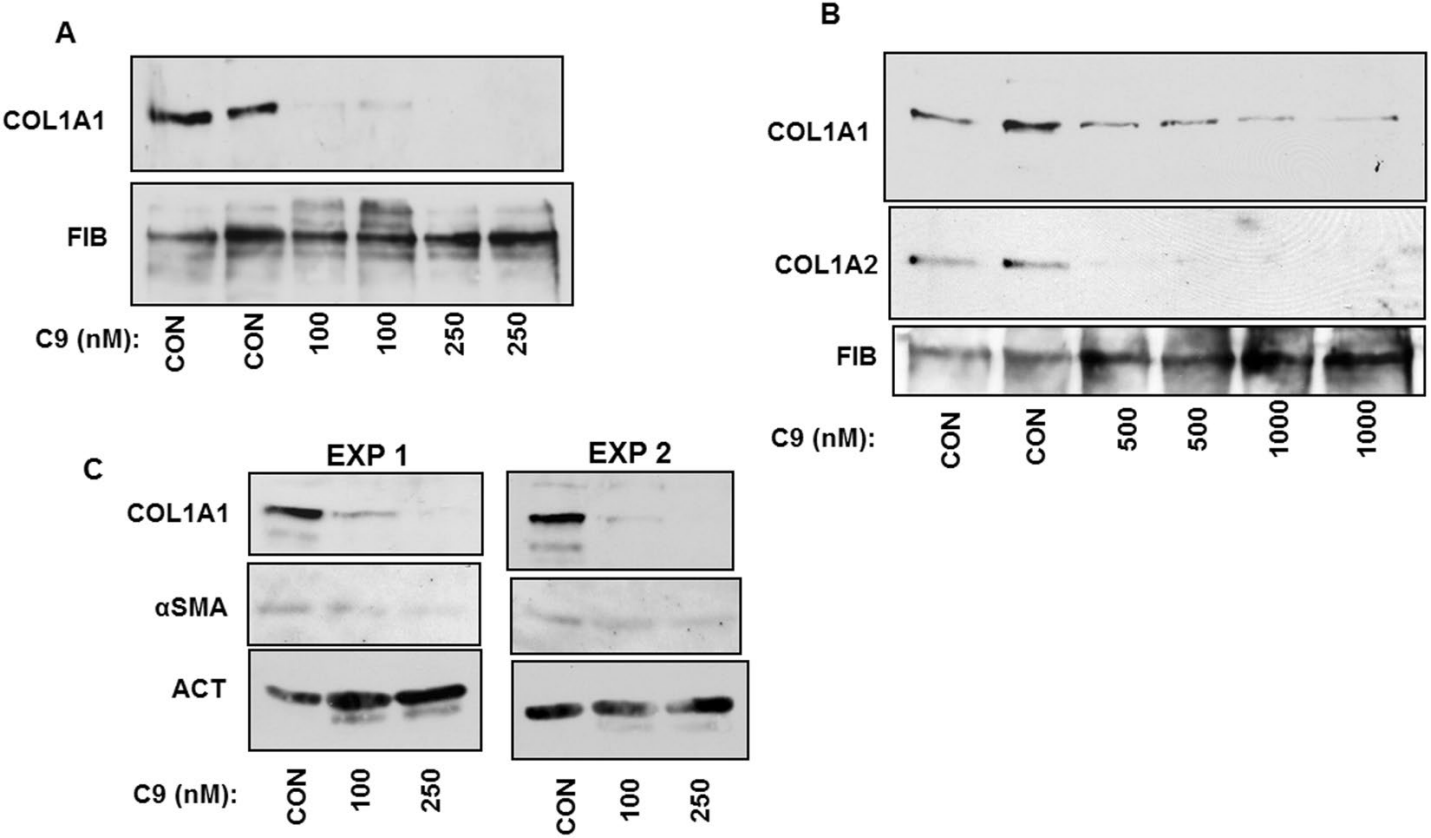
Suppression of type I collagen production by hepatic stellate cells (HSCs). (**A**) Inhibitory effect of C9 in HSCs during differentiation from quiescent into activated phenotype. Primary rat HSCs at day 3 in culture were treated with indicated concentrations of C9 and cellular medium was analyzed for accumulation of collagen α1(I) polypeptide (COL1A1) by western blot at day 5. Loading control, fibronectin (FIB). (**B**) Inhibitory effect in activated HSCs. HSCs at day 5 in culture were treated with indicated concentrations of C9 until day 7 and cellular medium was analyzed for collagen α1(I) (COL1A1) and α2(I) polypeptide (COL1A2) by western blot. FIB, fibronectin as loading control. (**C**) Effect on expression of α-SMA in two independent experiments. Primary rat HSCs at day 3 in culture were treated with indicated concentrations of C9 and cellular extract was analyzed at day 5 for expression of α-SMA, COL1A1 and actin (ACT) as loading control.

HSCs are liver cells which are activated in fibrosis to produce large quantities of type I collagen^14,35,36^. Profibrotic activation of HSCs can be mimicked *in vitro* by culturing the freshly isolated HSCs on uncoated plastic^37^. Culturing of freshly isolated rat HSCs for 3 days is sufficient to trigger activation and synthesis of type I collagen increases between day 3 and day 5 in culture. To assess the effect of C9 on collagen production during activation of HSCs we treated rat HSCs at day 3 with 100 nM and 250 nM of C9. The treatment was until day 5, when we analyzed the secretion rate of collagen α1(I) polypeptide into the cellular medium. The morphology and viability of HSCs did not change by the C9 treatment (Supplemental Fig. 3), however, the secretion of type I collagen was dramatically reduced (Fig. 4A). While control cells secreted significant amount of collagen α1(I) polypeptide at day 5, the cells treated with 100 nM of C9 secreted only tracing amounts, while for the cells treated with 250 nM the secretion was undetectable. Secretion of fibronectin was not affected, suggesting the specific effect of C9 on type I collagen.

To assess if C9 can inhibit type I collagen production by HSCs that have been fully activated, we cultured HSCs for 5 days and treated the cells with C9 from day 5 to day 7. With HSCs at day 7 we could measure α1(I) and α2(I) polypeptides of type I collagen in the cellular medium. In the initial experiments we did not see an effect at 100 nM and 250 nM of C9, so we increased the concentration to 500 nM and 1 µM. At these concentrations C9 inhibited secretion of both collagen polypeptides, without affecting the secretion of fibronectin (Fig. 4B). Thus, when HSCs had already progressed into a more activated phenotype, the higher concentrations of C9 were needed to suppress type I collagen production.

To assess the effect of C9 on other markers of HSCs activation we measured α-smooth muscle action (α-SMA) expression in cellular extracts of HSCs. We treated rat HSCs at day 3 with 100 nM and 250 nM of C9, lysed the cells at day 5 and assessed α-SMA expression by western blot (Fig. 4C). There was no difference in α-SMA expression with the C9 treatment, while collagen α1(I) polypeptide was dramatically reduced. This indicated that *in vitro* C9 specifically reduces production of type I collagen and does not affect α-SMA as a marker of activation of HSCs.

### Inhibitory effect of C9 in human lung fibroblasts (HLFs)

**Figure 5:**
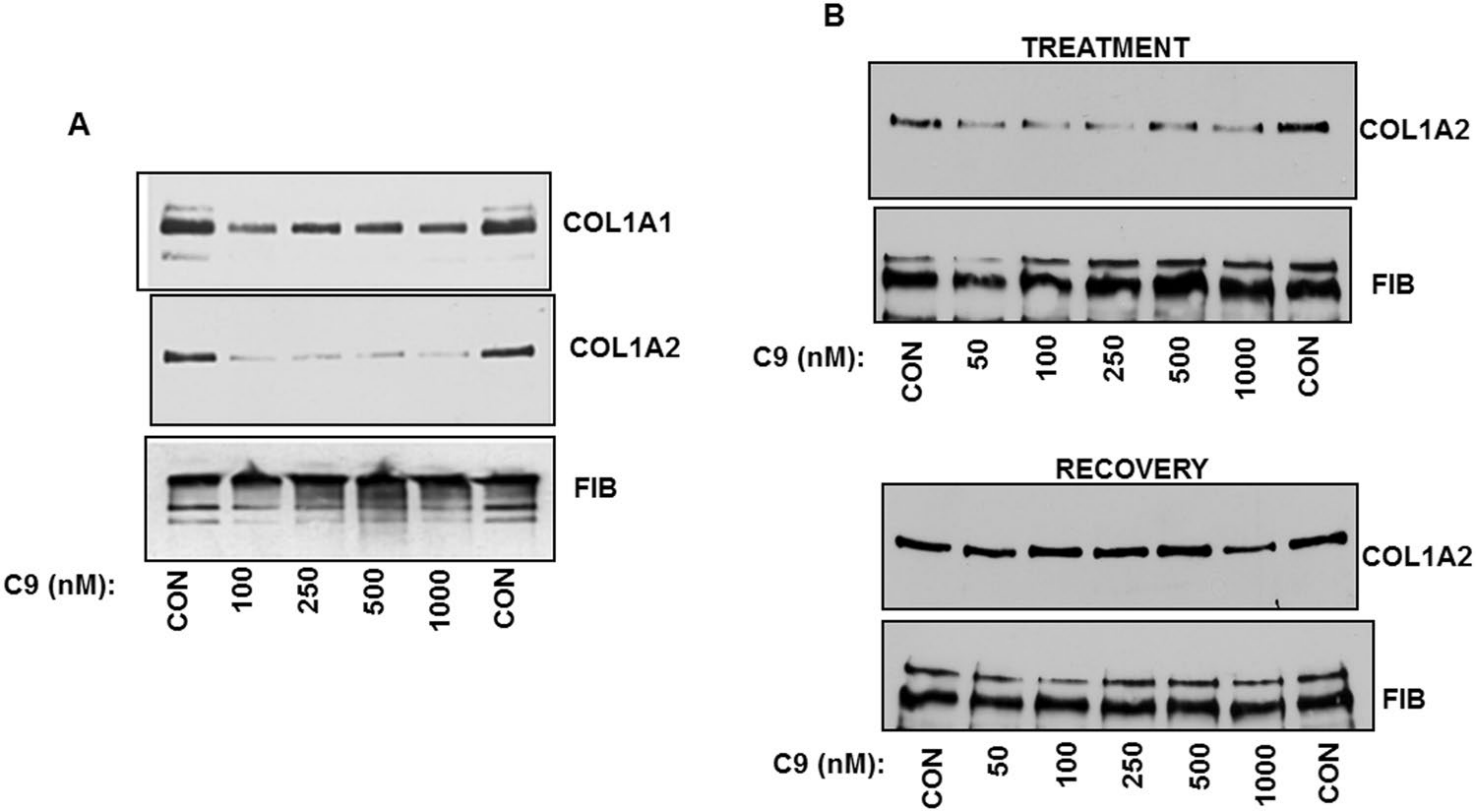
Reversible effect of C9 on type I collagen production. (**A**) Suppression of type I collagen production by HLFs. HLFs were treated with indicated concentrations of C9 overnight and cellular medium was analyzed for collagen α1(I) (COL1A1) and α2(I) polypeptide (COL1A2) by western blot. FIB, fibronectin as loading control. (**B**) Removal of C9 reverses the inhibitory effect. Upper panel: HLFs were treated with indicated concentrations of C9 overnight and cellular medium analyzed for collagen α2(I) polypeptide (COL1A2). Lower panel: C9 was washed off from the cells, the cells were incubated for additional 24 h and the medium was analyzed for collagen α2(I) polypeptide (COL1A2). FIB, fibronectin as loading control.

To assess if C9 has an inhibitory effect in other collagen producing cells we analyzed the effect in HLFs. The HLFs line used here produces high level of type I collagen^33,38^. In HLFs C9 at 100 nM reduced secretion of collagen α1(I) and α2(I) polypeptide by ~50% and ~80%, respectively, however, no further decrease was observed with increasing concentrations up to 1 μM (Fig. 5A). Secretion of fibronectin was unchanged, indicating that the general protein secretion machinery remained intact. Of note is that the dose of C9 (100 nM) which decreased type I collagen was the same as that which abolished binding of LARP6 in the extracts of these cells (Fig. 3). This result indicated that suppression of LARP6 binding activity in HLFs correlates with suppression of type I collagen production. The inability of higher doses of C9 to further inhibit type I collagen may suggest that HLFs also maintain LARP6 independent synthesis.

HLFs maintain relatively constant collagen production when cultured; this allowed us to assess if the effect of C9 is reversible and if wash off of C9 would result in restoration of type I collagen secretion. To this goal, we analyzed secretion of α2(I) polypeptide from HLFs treated with C9 and 24 h after the wash off of C9. Figure 5B, top panel shows that C9 reduced secretion of α2(I) polypeptide after an overnight incubation. The same cells were then washed and incubation continued for additional 24 h. 24 h after the C9 wash off secretion of α2(I) polypeptide returned to the control levels in cells treated with all concentrations of C9, except 1 µM (Fig. 5B, bottom panel). At 1 µM there was a partial recovery, either due to incomplete removal of C9 or to some long lasting effects of high C9 concentrations.

### Inhibition of profibrotic response in *ex vivo* model of hepatic fibrosis

**Figure 6:**
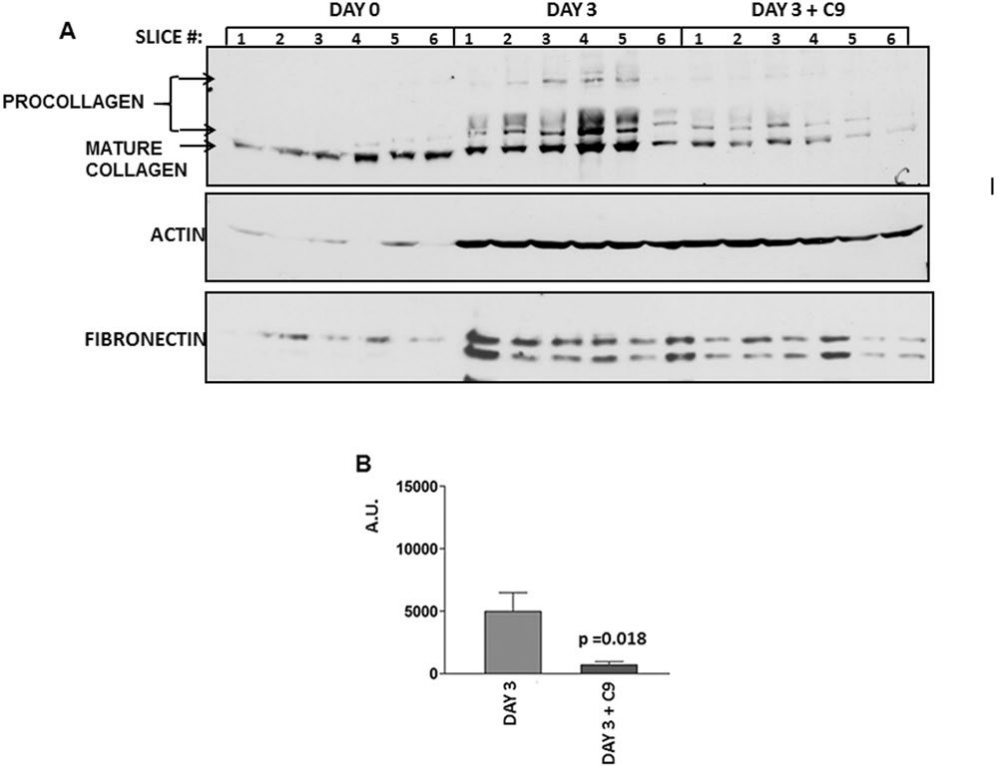
Suppression of profibrotic response in precision cut liver slices. (**A**) Analysis of procollagen accumulation in slices. Freshly prepared rat liver slices (day 0, slices 1–6), the slices cultured for 3 days without treatment (day 3, slices 1–6) and the slices treated with 1 μM of C9 from day 0 to day 3 (day 3 + C9, slices 1–6) were homogenized and type I collagen analyzed by western blot. Arrow indicates mature collagen and double arrow indicates nascent procollagen. Loading controls, actin and fibronectin. (**B**) Quantification of results. Intensity of bands representing procollagen was measured by densitometry from the slices shown in A and normalized to the actin band and plotted as arbitrary units (A.U.) P = 0.0184 by unpaired t test, error bars: +−SEM.

Culturing precision cut liver slices is well established *ex vivo* model of hepatic fibrosis^39–41^. After cutting, the liver slices initiate profibrotic response due to the injury of cutting. Slices can be cultured up to 3 days, after which their viability decreases, but within this time period a profibrotic response can be detected as appearence of the newly synthesized, nascent, procollagen^42^. Nascent type I procollagen has higher molecular weight than fully processed mature collagen and can be distinguished by western blots. We used this property to assess inhibitory effect of C9 on the profibrotic response of the slices. We cultured untreated slices or treated freshly cut liver slices with C9 at 1 µM for three days, homogenized the slices and measured procollagen by western blot. Figure 6A shows a result of six independent slices. The results with 18 additional slices are shown in the Supplemental Fig. 4. When analyzed, freshly cut slices contained only the pre-existing, mature type I collagen normally present in the liver (Fig. 6A, day 0, slices 1–6). When incubated for three days without treatment, the slices initiated synthesis of nascent procollagen, as evidenced by the appearance of higher molecular weight procollagen (day 3, slices 1–6). However, if the slices were treated with C9 for three days, the amount of nascent procollagen was significantly reduced, indicating attenuation of the response (day 3 + C9, slices 1–6). Expression of actin and fibronectin are shown as loading controls. Figure 6B shows quantification of procollagen in day 3 untreated and C9 treated slices from the experiment in Fig. 6A. The results from multiple experiments (Supplemental Fig. 4) indicated that C9 significantly reduced the ability of liver slices to initiate procollagen type I production.

To assess if expression of α-SMA in the slices was affected by C9 treatment, we measured α-SMA expression by western blot. Supplemental Fig. 5A shows a representative blot and Supplemental Fig. 5B shows quantification of α-SMA expression in 16 untreated and in 16 C9 treated slices. Similarly to the result of HSCs, there was no effect of C9 on α-SMA expression, suggesting that the C9 inhibition was specific for type I collagen. The results with precision cut liver slices prompted us to test C9 in animal models of hepatic fibrosis.

### Prophylactic effect in ethanol/CCl_4_ model of hepatic fibrosis

**Figure 7:**
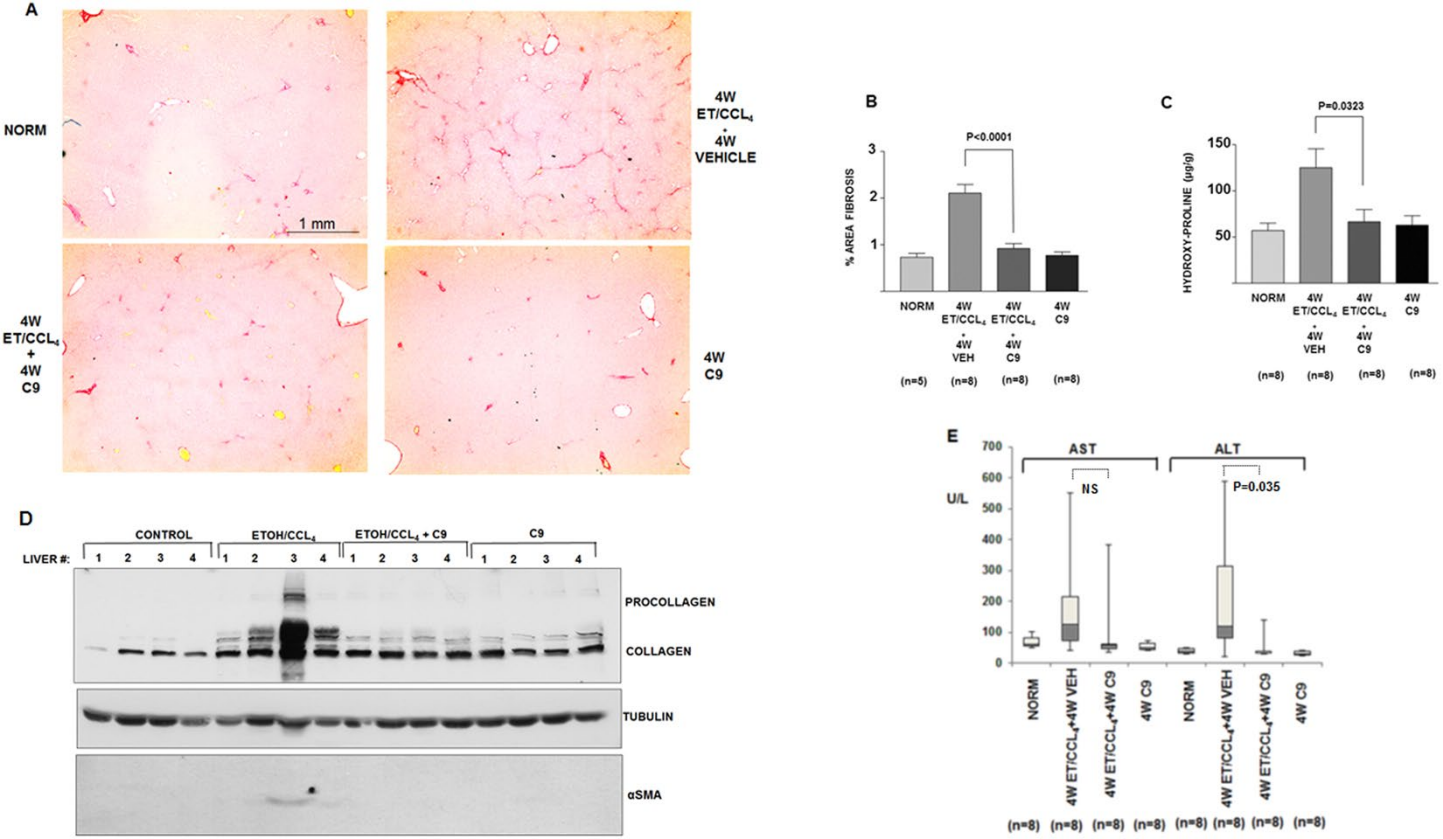
Prophylaxis of hepatic fibrosis by C9. (**A**) Representative images of Sirius red staining of liver sections (additional images are shown in the supplemental material). Upper left panel: normal liver. Upper right panel: fibrosis after 4 weeks in untreated animal. Lower left panel: fibrosis after 4 weeks in C9 treated animal. Lower right panel: liver of an animal receiving only C9 for 4 weeks. The scale bar (1 mm) is shown in the first image, all other images were taken under the same magnification. (**B**) Quantification of fibrosis. Left panel: morphometric quantification of fibrosis area from Sirius red stained histological slides. Significant reduction of fibrosis between nontreated and treated animals is indicated (p < 0.0001, unpaired t test). (**C**) Determination of hydroxy-proline content of the livers. Hydroxyproline was measured by colorimetric assay in duplicate and plotted (p = 0.0323, unpaired t test). Number of animals in each cohort is specified. Error bars: +−SEM. (**D**) Measurement of type I collagen and α-SMA by western blot. Liver samples were homogenized and total proteins were analyzed by western blot. Procollagen is indicated by bracket. α-smooth muscle actin (αSMA) was analyzed in the same samples and tubulin (TUB) is shown as loading control. (**E**) Aspartate aminotransferase (AST) and alanine aminotransferase (ALT) were measured in plasma of the indicated cohorts of animals. Box plots showing median, first quartile, third quartile and minimal and maximal values are shown.

Liver fibrosis was induced in Wistar rats by feeding the animals 5% ethanol in drinking water in combination with low dose CCl_4_ injections^43–45^. After four weeks of this treatment the control animals showed moderate fibrosis; Sirius red staining of a representative liver at 4x magnification is shown in Fig. 7A, upper right panel. The histology of seven additional livers is shown in Supplemental Fig. 6A. In the treated cohort the treatment with 1 mg/kg of C9, i.p., daily, started at the same time as the fibrosis induction. The dose was selected based on the efficacy of C9 in precision cut liver slices and based on its solubility in the vehicle used for injections. After four weeks, 1 mg/kg of C9 prevented fibrosis development in all animals (Fig. 7A, lower left panel; Supplemental Fig. 6B). We also administered C9 without fibrosis induction to assess the effect of the drug alone (Fig. 7A, lower right panel; Supplemental Fig. 6C). C9 administration alone did not cause any change in liver histology.

Using morphometric scanning of Sirius red stained histology slides from 8 animals in untreated group (vehicle) and treated group (C9) we determined the percentage of liver area affected by fibrosis (Fig. 7B). The C9 treated animals showed significantly less fibrosis (p < 0.001) compared to the nontreated animals. Figure 7C shows determinations of hydroxyproline, as a biochemical measure of liver collagen content^46^. In this assay there was also significant difference between the untreated and treated animals (p < 0.001).

Figure 7D shows western blot of expression type I collagen and α-SMA in liver extracts of the same animals; additional results are shown in Supplemental Fig. 6D. Type I collagen was highly upregulated in vehicle receiving animals, and presence of procollagen in the liver was evident. Treatment with C9 inhibited upregulation of procollagen, consistent with the absence of fibrosis in these animals and normal levels of hydroxyproline. α-SMA expression was detected in the animals receiving vehicle, but it was undetectable in animals treated with C9. This may suggest that *in vivo* C9 have suppressed α-SMA as a marker of HSCs activation, however, this effect may be a consequence of reduced type I collagen deposition. Lower amount of type I collagen in the extracellular matrix reduces its stiffness and stiffer extracellular matrix promotes activation of HSCs *in vivo*^47–50^.

Aminotransferases AST and ALT were also measured in the plasma and the result is shown in Fig. 7E. The levels of AST and ALT were on average lower in C9 treated animals than in vehicle treated animals, although there was a great overlap of the readings. This indicates that the hepatocellular injury was not substantially mitigated by C9. From this study we concluded that C9 at 1 mg/kg is highly effective in preventing ethanol/CCl_4_ induced liver fibrosis.

### Therapeutic effect in ethanol/CCl_4_ model of hepatic fibrosis

**Figure 8:**
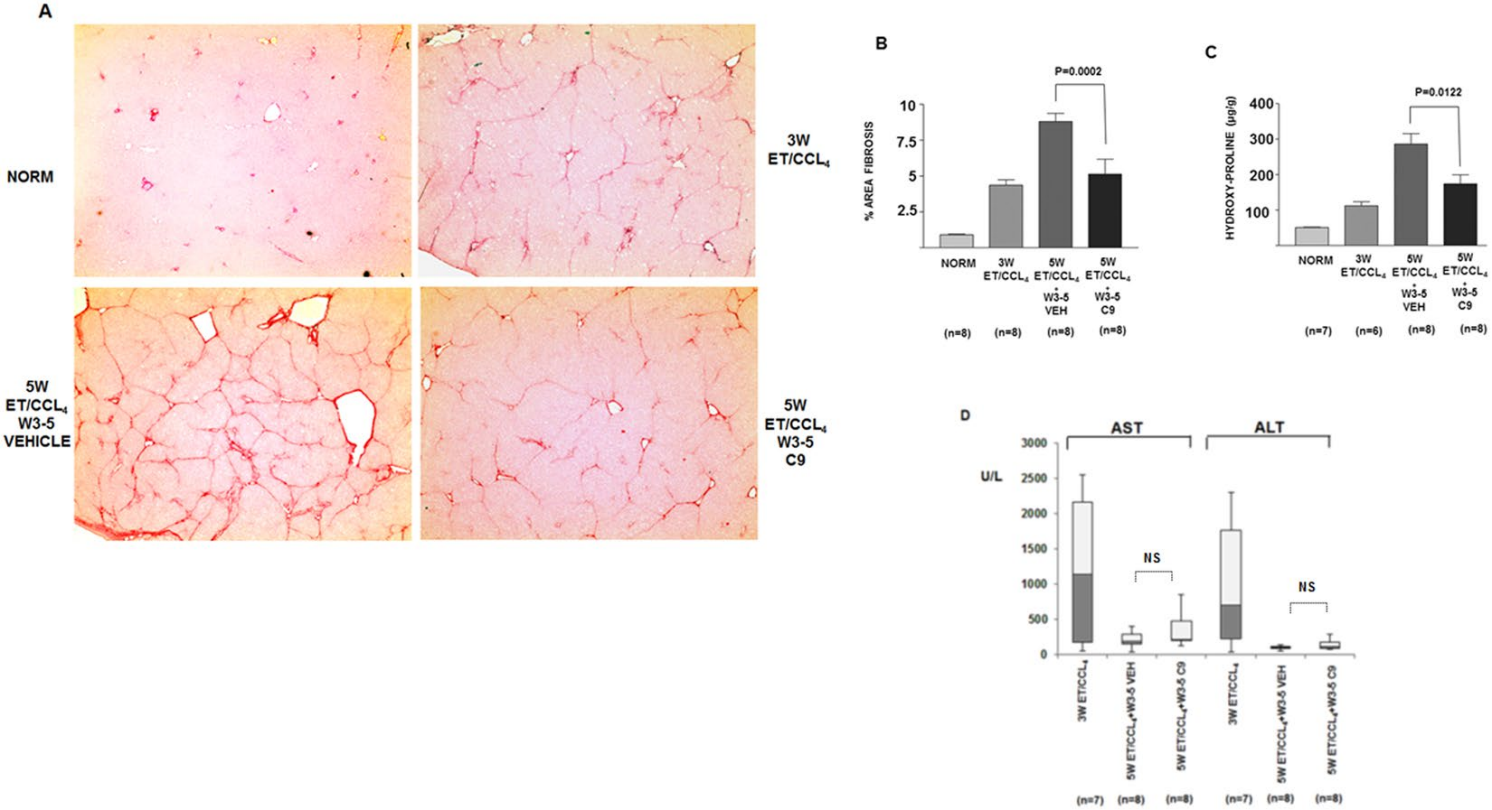
Treatment of CCl4/ethanol induced hepatic fibrosis by C9. (**A**) Representative images of Sirius red staining of liver sections, the same magnification as in Fig. 7 (additional images are shown in the supplemental material). Upper left panel: normal liver. Upper right panel: fibrosis after 3 weeks of CCl4/ethanol administration. Lower left panel: fibrosis after 5 weeks of CCl4/ethanol administration in an untreated animal. Lower right panel: fibrosis after 5 weeks of CCl4/ethanol administration in an animal treated from week 3 to week 5 with C9. (**B**) Quantification of fibrosis. Left panel: morphometric quantification of fibrosis area from Sirius red stained histological slides. Significant reduction of fibrosis between nontreated and treated animals is indicated (p = 0.0002, unpaired t test). (**C**) Determination of hydroxy-proline content of the livers (p = 0.0122, unpaired t test). Number of animals in each cohort is specified. Error bars: +−SEM. (**D**) Aspartate aminotransferase (AST) and alanine aminotransferase (ALT) were measured in plasma of the indicated cohorts of animals. Box plots showing median, first quartile, third quartile and minimal and maximal values are shown.

To extend the validation of C9 in therapeutic models of hepatic fibrosis, we first induced liver fibrosis in rats by ethanol/CCl_4_ treatment for 3 weeks. At this point the animals developed moderate fibrosis as shown in Fig. 8A, upper right panel and in Supplemental Fig. 7A. This served to assess the degree of fibrosis which would be expected at the onset of therapy. In the study we started the daily therapy with 1 mg/kg of C9, i.p. after 3 weeks of fibrosis induction and the C9 treatment lasted for two weeks, during which the ethanol/CCl_4_ application was continued. Without C9 treatment the fibrosis progressed from week 3 to week 5, with bridging and formation of cirrhotic nodules (Fig. 8A, lower left panel, Supplemental Fig. 7B), however, treatment with 1 mg/kg of C9 for the final two weeks resulted in less fibrosis seen on histology (Fig. 8A, lower right panel, Supplemental Fig. 7C). Quantification of fibrosis, either as morphometric scanning of histology slides (Fig. 8B) or as hydroxyproline measurements (Fig. 8C) between untreated and treated animals revealed that it was significantly reduced (p < 0.01).

Aminotransferase levels were very high after 3 weeks of ethanol/CCl_4_ treatment, suggesting significant liver damage in the first weeks of ethanol/CCl_4_ treatment (Fig. 8D). However, AST and ALT levels decreased by week 5 and there was no difference in vehicle and C9 treated animals.

### Prophylactic effect in early fibrosis induced by bile duct ligation

**Figure 9:**
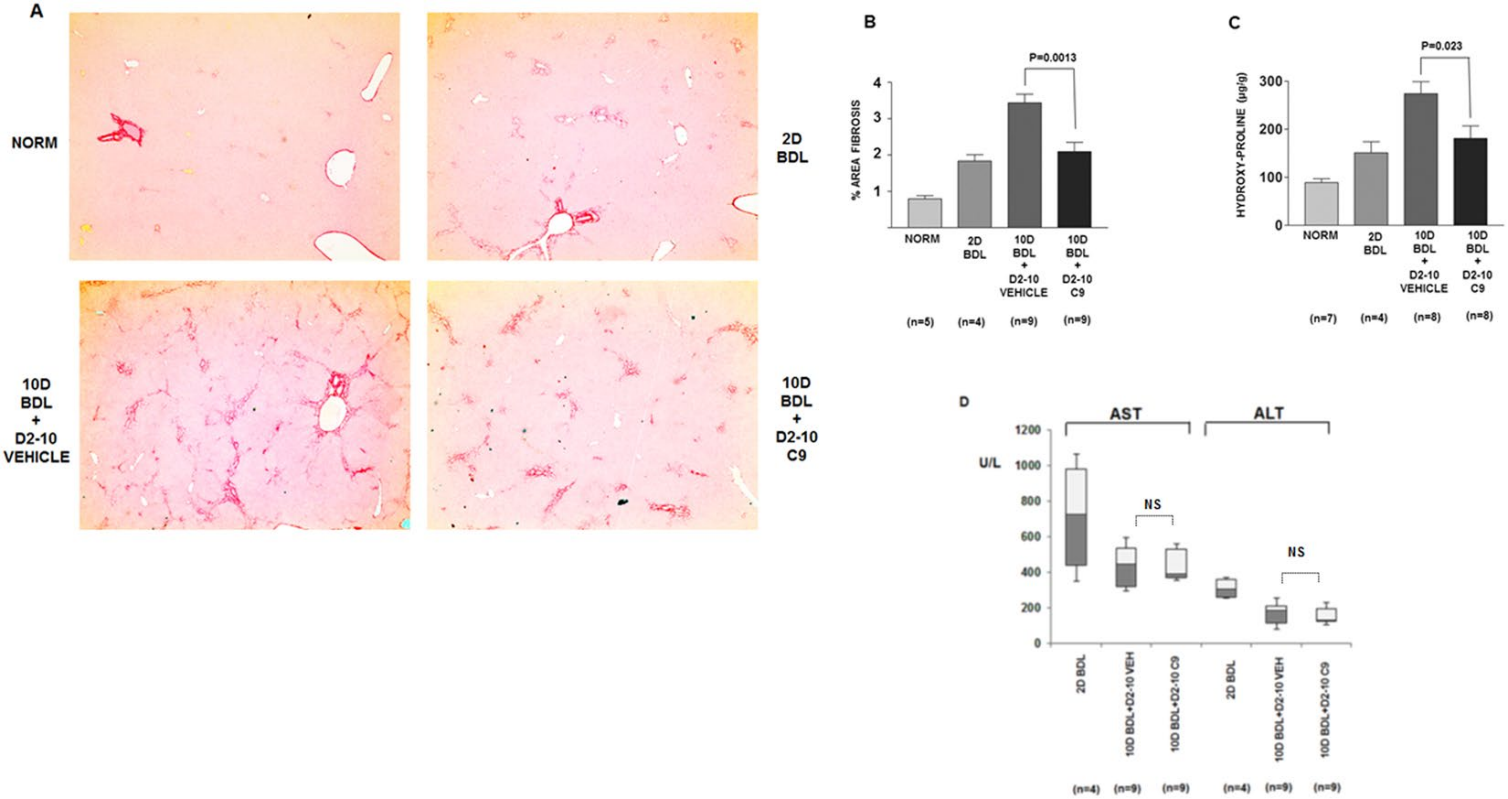
Prevention of early fibrosis induced by bile duct ligation. (**A**) Representative images of Sirius red staining of liver sections, the same magnification as in Fig. 7 (additional images are shown in the supplemental material). Upper left panel: normal liver. Upper right panel: fibrosis 2 days after bile duct ligation. Lower left panel: fibrosis 10 days after bile duct ligation in an untreated animal. Lower right panel: fibrosis 10 days after bile duct ligation in an animal treated from day 2 to day 10 with C9. (**B**) Quantification of fibrosis. Left panel: morphometric quantification of fibrosis area from Sirius red stained histological slides. Significant reduction of fibrosis between nontreated and treated animals is indicated (p = 0.0013, unpaired t test). (**C**) Determination of hydroxy-proline content of the livers (p = 0.023, unpaired t test). Number of animals in each cohort is specified. Error bars: +−SEM. (**D**) Aspartate aminotransferase (AST) and alanine aminotransferase (ALT) were measured in plasma of the indicated cohorts of animals. Box plots showing median, first quartile, third quartile and minimal and maximal values are shown.

Bile duct ligation is another standard model to induce hepatic fibrosis. When common bile duct is ligated in rats, early biliary fibrosis develops within several days^51^. This initial fibrosis is mild, but allows testing of the prophylactic effect. In our study the rats showed first signs of periportal fibrosis two days after the bile duct ligation (Fig. 9A, upper right panel, Supplemental Fig. 8A), therefore, to test the prophylactic potential of C9 we initiated the treatment at day 2 and continue the treatment until day 10. In untreated rats fibrosis progressed between days 2 and 10 (Fig. 9A, lower left panel, Supplemental Fig. 8B), but in rats receiving 1 mg/kg of C9 the fibrosis progression was inhibited (Fig. 9A, lower right panel, Supplemental Fig. 8C). The fibrosis was quantified between nontreated and treated animals by morphometric analysis (Fig. 9B) and by hydroxyproline measurements (Fig. 9C) and was significantly attenuated (p < 0.05). Based on these results we concluded that in early development of biliary fibrosis C9 compound has a significant effect in preventing the progression of fibrosis.

In the BDL model the aminotransferase levels were higher at 2 days after BDL and then at day 10. There was no difference in vehicle and C9 treated animals at day 10 (Fig. 9D), again suggesting that C9 has no effect on the degree of hepatocellular injury.

### Toxicogenetics analysis

To assess toxicity associated with administration of C9 we administered 10 mg/kg of C9, i.p., daily in 6 rats, while the vehicle was given to 2 rats. After 4 weeks the liver RNA was extracted and we also extracted RNA from 4 untreated livers for comparison of gene expression. The transcriptome was analyzed by RNA sequencing of all 10 samples.

We found 42 genes expression of which was significantly altered (FDR adjusted p value of <0.05) in livers of animals treated with C9, when compared to six control animals (Supplemental Table 1). To gain insight into the function of the genes that were differentially expressed between control and drug-treated livers, we examined gene ontology (GO) and KEGG pathway enrichment. We supplied 42 differentially expressed genes to WebGestalt (Zhang *et al*., 2005; Wang *et al*., 2013) and 31 genes were unambiguously associated with a gene ID in the database. As this is a small number of genes, there was no significant enrichment of KEGG pathways. The GO analysis showed that there is one significantly enriched GO process, “regulation of serine-type endopeptidase”. Three genes (Spink3, Spink1, and LOC100911558) were enriched for this GO process. In addition, two other genes, that were not associated with a gene ID in this search, have cysteine-type endopeptidase inhibitor activity (Kng1l1 and Kng2). These genes were all up-regulated in the drug-treated group. No significantly enriched GO processes were found that contain genes that were down-regulated in the drug-treated animals. However, Ptma, which was down-regulated in the drug-treated animals, is involved in negative regulation of cysteine-type endopeptidase activity.

Taken together, these analyses suggest that multiple genes that inhibit activity of serine and cysteine peptidases were differentially expressed in the drug-treated livers. Additional experiments will be needed to determine if this reflects a response to increased proteolytic activity induced by drug treatment. No genes associated with apoptosis, necrosis, cell proliferation, autophagy, oxidative stress or mitochondrial dysfunction were detected, suggesting the absence of overt hepatotoxic effect.

## Discussion

Fibrosis is a chronic disease that requires long term treatment, however, most antifibrotic drugs being developed lack the specificity towards type I collagen, which is the major component of the superfluous extracellular matrix^52,53^. Recently developed antibody against LOXL2 (enzyme involved in crosslinking of collagens) showed promising results in reducing fibrosis^10,11^, but the affordability and the side effects in long term application due to the effect on all collagen types and on the role of LOXL2 in transcription^54^ are of concern. Ideally, antifibrotic drugs would only target type I collagen biosynthesis within the fibrotic lesion and leaving the constitutive type I collagen synthesis unaffected. The discovery that binding of LARP6 to the 5′SL sequence of type I collagen mRNAs is critical for fibrosis development^30^ opened the possibility that profibrotic production of type I collagen can be specifically targeted. This work is the first attempt to find inhibitors of LARP6 binding to collagen mRNAs by screening of a chemical library.

The rationale to search for LARP6/5′SL RNA binding inhibitors came from studies on posttranscriptional regulation of type I collagen biosynthesis^17,20,23^. Type I collagen is composed of two α1(I) and one α2(I) polypeptides which fold into a heterotrimer. The high rate of type I collagen production in fibrosis requires coordination of translation of α1(I) and α2(I) polypeptides^18^, this process is regulated by binding of LARP6 to a secondary structure in the 5′ UTR of collagen mRNAs, the 5′SL^23,26^. The LARP6/5′SL interaction is specific for regulation of translation of type I collagen mRNAs, because the 5′SL is not found in other mRNAs. A similar structure is found in type III collagen mRNA, but it binds LARP6 with lower affinity^23^. Therefore, LARP6 can be regarded as the specific regulator of type I (and possibly type III) collagen. Importantly, the LARP6/5′SL interaction is dispensable for constitutive type I collagen synthesis, as evidenced by the developed knock in animal model^30^.

It is a common assumption that RNA-protein interactions are too complex to be disrupted by small molecules. However, multiple examples have been presented that it is possible to inhibit RNA-protein interactions by chemical compounds. The classic examples are aminoglycoside antibiotics^55^, the other examples include an inhibitor of HIV Gag interaction with the stem-loop 3 (SL3) of HIV genomic RNA^56^, an inhibitor of the interaction of HIV nucleocapsid protein NCp7 with SL3^57^, inhibitors of bacterial riboswitches and inhibitors of muscleblind-like 1 protein binding to CUG repeats of the DMPK mRNA^58^. This is the first report that it is possible to find small molecules that can inhibit progression of fibrosis by suppressing the binding of LARP6 to 5′SL. We showed that: 1. C9 inhibits the interaction of LARP6 and 5′SL RNA *in vitro* and in extracts of cells treated with the drug, 2. C9 alters the intrinsic fluourescence of LARP6 tryptophanes, suggesting that the compound induces a conformational change of LARP6, 3. C9 inhibits type I collagen production in HSCs undergoing activation *in vitro* at concentrations of 100–250 nM. 4. C9 suppresses profibrotic response in precision cut liver slices at 1 μM, 5. in animal models of hepatic fibrosis, significant inhibition of fibrosis has been observed when C9 was administered at 1 mg/kg.

Few considerations have to be kept in mind when interpreting the results of our animal models. First, the BDL model was of short duration, when only early biliary fibrosis had developed. In this setting, we could assess only the prophylactic effect of C9 in initiation of biliary fibrosis. To assess a therapeutic effect, additional studies requiring longer duration of fibrosis are needed. It has been reported that imatinib, a tyrosine kinase inhibitor, prevents early fibrosis, but not late stage fibrosis^59^. It is possible that C9 may have similar property. Second, the number of animals in our study was relatively small. Although significant effects on fibrosis were observed, including larger number of animals may have changed the result, because of the considerable biological variability in fibrosis response that may have not been represented in our study.

Taking into account the above caveats, we believe that the C9 compound represents a LARP6 inhibitor with promising antifibrotic activity and may represent a therapeutic lead for further development into a specific antifibrotic drug.

*In vitro*, C9 does not affect expression of α-SMA by HSCs, as a marker of their activation, just the expression of type I collagen (Fig. 4C). Likewise, in precision cut liver slices α-SMA expression is also not affected. This is consistent with the notion that C9 is collagen specific drug and not a compound that has an effect on other markers of HSCs activation. *In vivo*, activation of HSCs is augmented by the increased stiffness of the extracellular matrix in which they reside^47–50^. *In vivo*, it is possible that inhibition of type I collagen by C9 reduced the stiffness of extracellular matrix and as a result the expression of α-SMA was reduced, as was observed in the prophylactic model (Fig. 7D). C9 also does not affect the degree of hepatocellular injury, as assessed by measuring aminotransferases. These, results are expected as C9 has been discovered to be type I collagen specific compound.

Preliminary evidence suggests that C9 causes ubiquitination of LARP6 *in vivo*, possibly as a result of altering its conformation. We speculate that this his may explain much higher efficacy *in vivo* than *in vitro*; C9 can inhibit type I collagen production by HSCs and HLFs at 100 nM, while the effects on *in vitro* binding are seen at μM concentrations. In animal models, hepatic fibrosis was prevented or attenuated at doses of 1 mg/kg; efficacy at such a dose implies that the compound can be promising therapeutic agent.

A few functions of LARP6, other than regulation of type I collagen, have been described. They include regulation of muscle differentiation and integrin expression in differentiating myoblasts and differentiation of ciliated cells in embryonic development^60–63^. However, these functions do not involve binding of collagen mRNAs, therefore, it is not clear if they will be affected by LARP6 binding inhibitors.

We have not comprehensively tested toxicology and pharmacokinetics of C9. One pilot study indicated that C9 is orally bioavailable in rats; after an oral gavage of 10 mg/kg of C9 the maximal plasma concentration of 350 ng/ml was measured after 5 h (Supplemental Fig. 9). No adverse effects were observed after administering C9 at 10 mg/kg, i.p., daily, for 4 weeks in rats. Body weight gain and histology of major organs were indistinguishable from controls. Toxicogenetics is a powerful method to predict possible toxicity of the drug by analyzing changes in gene expression after administration of a drug^64,65^. We compared the transcriptome of livers of rats treated with 10 mg/kg of C9, i.p. daily for 4 weeks to vehicle treated and untreated livers. Out of 26,405 genes analyzed only 42 genes were differentially expressed by C9 treatment. Several of these genes encode for serine or cysteine protease inhibitors, suggesting a response to increased proteolytic activity in the liver, but this finding will require further evaluation. The fact that only 42 genes changed expression strongly argues that there had been no major alteration of cellular processes with C9 application and none of the identified genes was direct indicator of cellular toxicity or injury.

In conclusion, this is the first report describing a chemical compound with antifibrotic activity that acts as inhibitor of LARP6 binding to collagen mRNAs. Compounds of this class may be truly specific antifibrotic drugs and suitable for long term therapy. Further preclinical development of C9, particularly in long term BDL studies, and screenings for additional LARP6 inhibitors may lead to development of breakthrough drugs for treatment of hepatic and possibly other forms of fibrosis.

## Materials and Methods

### Preparation of recombinant LARP6

Construct for expression of LARP6 having only the minimal sequence required for binding 5′SL (amino-acids 72–303, designated here LARP6*) was described before^25^. pET15b plasmid harboring this construct was transformed into Roseta *E. coli* strain and the cells grown in terrific broth to OD_260_ of 0.8, after which the expression was induced by 1 mM IPTG and growth continued for 5 h at 25  C. His-tag LARP6* was purified by Ni-column according to manufacturer′s protocol (Invitrogen). The purified protein was dialyzed against PBS and its purity was estimated by SDS-PAGE and staining with Fast-blue. Typically, the purity was >90% after a single step purification.

### High throughput screening

5′ stem-loop RNA sequence of human collagen α1(I) mRNA was labeled at the 5′ end by fluorescein and was synthesized by Dharmacon (fl-5′SL RNA). 25.6 nM of LARP6* and 1 nM of fl-5′SL RNA were mixed in 100 mM NaCl, 10 mM Tris pH 7.5, 5 mM MgCl_2_, 0.25% NP-40 and 25 μL/well was dispensed into 384-well black microplates (Brand). 0.5 μl of the compounds from the ChemBridge Inc., DIVERSet-CL, code NT1174 chemical library (50,000 compounds) was added (final concentration 200 μM) and fluorescence polarization (FP) was read in Biotek H1 Synergy plate reader fitted with automated plate feeder. The mean value of FP of all wells (screen FP) and the standard deviation (SD) was calculated and a decrease of FP by >3 SD of the screen FP was considered a hit^32^. As control, FP readings of wells without compounds was also calculated.

### Titration of LARP6* binding to fl-5′SL RNA *in vitro*

For titration of fl-5′SL RNA with increasing concentrations of LARP6* 1 nM of fl-5′SL RNA was incubated with increasing concentrations of LARP6* in in 100 mM NaCl, 10 mM Tris pH 7.5, 5 mM MgCl_2_, 0.25% NP-40. The reactions were incubated for 10 min at RT in 384 well plates in total volume of 25 µl/well and FP was read in Biotek Synergy H1 plate reader. A blank FP without LARP6* was subtracted from the FP of the fl-5′SL/LARP6* reactions and corrected FP was plotted against LARP6* concentration. All reactions were performed in triplicates and apparent dissociation constants (Kd) were estimated by nonlinear curve fitting as the concentration which gives 50% saturation.

### Measurement of intrinsic fluorescence of LARP6 tryptophans

Unfolding of LARP6* was monitored by change in fluorescence emission spectrum of the protein after addition of increasing amounts of C9 (λex = 277 nm, λem = 280–500 nm; 5 nm slit widths) using a Cary Eclipse spectrophotometer (Agilent Technologies, Santa Clara, California). Increasing amounts of C9 were added to the cuvette containing sufficient amount of LARP6* to give intrinsic fluorescence of ~140 arbitrary units (A.U.) and fluorescent spectrum was recorded.

### Growing of HSCs and human lung fibroblasts

Isolation of HSCs was done in accordance to the guidelines and regulations of National Institute of Health, USA (NIH) for use of vertebrate animals in research. The experimental protocol for HSCs isolation was approved by the Animal Care and Use Committee (ACUC) of Florida State University; protocol number 1428. All methods were carried out in accordance with relevant guidelines and regulations. All experimental protocols were approved by the Florida State University ACUC committee.

Rat HSCs were isolated by perfusion of rat liver with 0.5 mg of pronase and 0.04 mg of collagenase per gram of animal weight, followed by centrifugation of the cell suspension over 20% Nykodenz gradient, as described^66^. After isolation, the cells were cultured in uncoated plastic dishes in DMEM supplemented with 10% fetal bovine serum (FBS) and antibiotics for 3 days, the medium was changed and C9 was added to the medium at indicated concentrations and incubated for 2 days. In some experiments HSCs were cultured for 5 days to become activated and then treated with C9 from day 5 to day 7.

Human lung fibroblasts (HLFs) were from the cell line hTERT-1604 (RRID:CVCL_C472), developed by Ouellette *et al*. in 2000 by immortalizing human fetal lung fibroblasts by expression of the catalytic subunit human telomerase reverse transcriptase (hTERT)^33,38^. HLFs were grown until 80% confluency in DMEM/10% FBS and C9 was added to the medium for 18 h.

### Gel mobility shift assay

Cell extracts were prepared by lysis of cells in 140 mM KCl, 10 mM MgCl_2_, 0.5% NP-40 and equal amounts (20 μl) were used for gel mobility shift. The 5′SL RNA probes were prepared by *in vitro* transcription in the presence of ^32^P-UTP from templates containing 5′SL sequence of human collagen α1(I) mRNA. The amount of probe used in the reactions was typically 4 ng and the binding reaction contained 2 µg of yeast t-RNA and 1 μg of total liver RNA to suppress nonspecific binding^25^. After incubation at RT for 30 min the reactions were resolved on 6% native gels. Detection of the bands was by autoradiography or by phosphoimaging.

### Western blots

For measuring collagen secretion into the cellular medium, cells were washed with serum free medium and incubated in serum free medium for 3 h. The medium was collected and collagen was directly measured by western blot by loading 50–100 μL of the medium onto 7.5% SDS-PAGE gels^27,67^. Western blots were performed with 1:1000 dilutions of the antibodies. The antibodies used were: anti-collagen α1(I) from Rockland (600-401-103), anti-collagen α2(I) from Santa Cruz Biotechnology (sc-8786), anti-fibronectin from BD Transduction Laboratories (610523 and 610077). When cell extracts were analyzed anti-actin from Abcam (ab8227), anti-LARP6 from Novus Biologicals (H00055323) antibodies were used.

### Preparation of precision cut liver slices

Precision cut liver slices were prepared as described^39–41^. The method was carried out in accordance with relevant guidelines and regulations, Animal Care and Use Committee of Florida State University; protocol number 1123. Briefly, fresh rat liver was placed into ice cold organ processing solution (Mediatech, Inc., 98-451-CV) and cut in the same solution into slices of 8 mm diameter and 350 μM thick using Krumdieck microtome. Slices were cultured in 6-well plates (1 slice per well) in DMEM supplemented with 10% FBS, 0.4 μg/ml dexamethasone, 50 μg/ml ascorbic acid, 0.5 μg/ml insulin under 100% oxygen for 3 days. C9 was added to the medium of slices at 1 μM immediately after the preparation. For determination of procollagen, slices were homogenized either fresh or after 3 days of culturing in 150 mM NaCl, 1% SDS, 10 mM Tris pH 7.5, clarified by centrifugation and 200 μg of total protein was analyzed by western blot using anti-collagen α1(I) antibody from Rockland (600-401-103). For quantification of procollagen, western blots were densitometrically scanned and the intensity of bands representing procollagen was normalized to the intensity of actin band. Unpaired t test was used to compare the groups (N = 6). α-SMA was analyzed using and α-SMA antibody from Sigma (A2547) anti-BHMT antibody H-46 from Santa Cruz (sc-134648).

### Animal models of hepatic fibrosis

Animal studies were performed according to the guidelines and regulations of National Institute of Health, USA (NIH) for use of vertebrate animals in research. The experimental protocols were approved by the Animal Care and Use Committee (ACUC) of Florida State University; protocols number 1417 and 1119. All animal procedures were done according to the guidelines of the Florida State University ACUC committee.

For the prophylactic ethanol/CCl_4_ model Wistar rats were randomized upon arrival and received standard chow diet with ab libitum access to water and food for 5 days. At the start of the study, drinking water was replaced by 5% ethanol as the only source of liquid, except for the cohort receiving C9 only. Three cohorts of rats, males, 150–200 g, 8 animals per cohort, were established as follows: the non-treated cohort, received 5% ethanol liquid + CCl_4_ injections + vehicle injections; the treatment cohort, received 5% ethanol liquid + CCl_4_ injections + C9 injections; the C9 cohort, received C9 injections only. The liquid intake and body weight was recorded daily. Carbon tetrachloride (CCl_4_) was administered intraperitoneally (i.p.) at 0.5 µl/g of CCl_4_ in mineral oil twice a week for 4 weeks^43–45^. C9 was obtained from ChemBridge, Inc. (>99% pure) and was dissolved in 200 uL of 10% DMSO, 10% Cremaphor (Sigma) and 80% PBS and injected at daily dose of 1 mg/kg starting at the beginning of the study. After 4 weeks animals were sacrificed and livers were analyzed.

For the therapeutic ethanol/CCl_4_ model Wistar rats were grouped into the following cohorts of 8 animals per cohort: the baseline cohort, received 5% ethanol liquid + CCl_4_ injections for 3 weeks; the non-treated cohort, received 5% ethanol liquid + CCl_4_ injections for 5 weeks + vehicle injections in the last 2 weeks; the treatment cohort, received 5% ethanol liquid + CCl_4_ injections for 5 weeks + C9 injections in the last 2 weeks.

For prophylactic bile duct ligation model common bile duct was ligated in Sprague-Dawley rats; the procedure was performed by Charles River Laboratories where the animals were purchased. The ligated rats were divided into three cohorts; the baseline cohort, 2.5 days with bile duct ligation, N = 5; the non-treated cohort, 10 days with bile duct ligation + vehicle injections from day 2.5–10, N = 9, and the treatment cohort, 10 days with bile duct ligation + C9 injections from day 2.5–10, N = 9.

Unpaired t test was used to calculate the statistical significance between nontreated and treated groups of animals in each study.

### Histological analysis of fibrosis

Liver tissue was fixed in 10% formalin in PBS, embedded in paraffin and 10 µM thick slices were placed on microscope slides and stained with H&E and Sirius red. Images of Sirius red stained slides at 4x magnification were taken by EVOS XL Core microscope. To quantify fibrosis Sirius red images were converted into black and white images and area of fibrosis (Sirius red, dark pixels) was measured by Image J software. Percent fibrosis was calculated by dividing Sirius red pixels by the total pixels.

### Determination of hydroxy-proline

Hydroxy-proline measurement was performed in duplicate on each liver sample. Liver samples were hydrolyzed at 20 mg/100 μL of 6 N HCl for 18 h at 120 C, neutralized with 12 N NaOH and colorimetric reaction with chloramine-T was developed as described^68^. Calibration curve obtained with pure hydroxy-proline was used to determine the concentrations from the OD_560_.

### Aminotransferase measurement

ALT and AST were measured in duplicate in plasma samples by the kinetic method^69^. Box plots showing median, first quartile, third quartile and minimal and maximal values are presented.

### RNA isolation and RNA-Seq library preparation

Total liver RNA was isolated using Trizol reagent (Sigma). RNA-Seq libraries were prepared using NEBNext sequencing reagents. mRNA enrichment was performed with the NEBNext Poly(A) mRNA Magnetic Isolation Module (New England Biolabs, Catalog E7490S) and libraries were prepared using NEBNext Ultra RNA library kit for Illumina sequencers (NEB, Catalog E7520S). A unique 6 nucleotide index was incorporated into each library (NEB, Catalog E7335S) in order to combine (multiplex) multiple libraries in one sequencing lane. The concentration of each library was estimated with qPCR (KAPA-PCR) using Illumina sequencing primers (KAPA Biosystems catalog #KK4835), and average fragment length was determined with a bioanalyzer high sensitivity DNA chip (Agilent Technologies, catalog #5076-4626). Six control and 6 drug-treated liver libraries were generated for each condition and 12 pM of each multiplexed library (six per sequencing lane) were sequenced on an Illumina HiSeq 2500 located in the Translational Science Laboratory at the College of Medicine, Florida State University.

### RNA-Seq data analysis

Initial quality control (QC) analysis of each sequenced library was performed using fastQC. Adapter trimming was performed with Trimmomatic^70^, using the TruSeq 3-SE.fa adapter sequence file provided with the program. A minimum length of 50 bases (MINLEN:50) was chosen as a cutoff for each sequenced unit (read) during the adapter removal. The trimmed sequencing reads were aligned and mapped using Tophat 2.0.13^71^ to the rat genome (genome release Rattus norvegicus 5 or Rnor_5.0). A General Transfer Format (GTF) file was provided to Tophat (–GTF option) for the purpose of gene annotation. Following mapping with Tophat, reads were filtered with Samtools^72^ and only unique reads that mapped to a single gene were used for further analysis. The unique reads were used to generate counts for each annotated gene using EasyRNASeq^73^. The Rattus_norvegicus.Rnor_5.0.73.gtf file was used for annotation and “geneModels” was used as the summarization option. Statistical analysis on count tables for male versus female comparisons of each strain was performed using DESeq2^73^ to determine statistically significant differentially expressed genes. A false discovery rate, (FDR) adjusted p-value of <0.05, using the Benjamini-Hochberg adjustment for multiple testing, is considered significant in our study^74^.

### Gene Enrichment and Network Analysis

WebGestalt^75^ was used to determine gene ontology (GO) enrichment. For enrichment analysis, the Rattus norvegicus genome was used as the reference set to obtain significantly enriched (FDR adjusted p-value < 0.05) ontologies. The statistical test was hypergeometric and the Benjamini-Hochberg FDR method was used for multiple testing adjustment.

### Equipment and settings

The gel images shown in Figs 3, 4, 5, 6 and 7 and in Supplemental Figs 4 and 6 were obtain from X-ray films exposed to autoradiography gels or to western blot membranes. X-ray films were scanned and trimmed by Photoshop program. Signals of western blots in Supplemental Fig. 5 were acquired by BioRad ChemiDoc MP imaging system. Minimal adjustment of the brightness and contrast was made. Histology images shown if Figs 7, 8 and 9 and in Supplemental Figs 6, 7 and 8 were taken by EVOS XL CORE imaging microscope at 4x magnification with cool color balance. The images were adjusted to similar brightness by Photoshop program.

## Electronic supplementary material

supplementary information
