# Supplementary material for: Discovery and evaluation of inhibitor of LARP6 as specific antifibrotic compound
Source: Sci Rep. 2019 Jan 23;9:326. doi: 10.1038/s41598-018-36841-y (PMC6344531; doi:10.1038/s41598-018-36841-y)
Supplement: Supplementary file 1 — supplementary information [file 41598_2018_36841_MOESM1_ESM.pdf]

# Discovery and evaluation of inhibitor of LARP6 as specific antifibrotic compound

**Branko Stefanovic[1*], Zarko Manojlovic[2], Cynthia Vied[3], Crystal-Dawn Badger[3,4], Lela Stefanovic[1]**

## Supplemental figure legends.

Supplemental figure 1. Statistics of high throughput screen. The mean of FP readings of controls without compounds (NO COMP) and the mean of FP readings of all wells receiving the compounds (FP SCREEN) with +- 1SD error bars is shown. Blank reading was not subtracted from these values because it was the same throughout the screen. The cut off value of FP SCREEN – 3SD is indicated; the compounds which showed FP lower than this limit were considered hits. As an example, the FP reading of C9, C10 and C11 compounds is shown.

Supplemental figure 2. Quenching of intrinsic tryptophan fluorescence of LARP6* by C9. A. Stern/Volmer plot of data from figure 2C, left panel. B. Titration of C9 into PBS. C.Titration of C9 into GST-LARP4 fusion protein. D. Titration of C9 into GST protein.

Supplemental figure 3. Morphology of primary rat HSCs after treatment with C9. Freshly isolated rat HSCs were cultured for 3 days, treated with 100 nM of C9 from day 3 to day 5 and images were taken.

Supplemental figure 4. Effect of C9 in precision cut liver slices. A. An independent preparation of liver slices analyzed for pro-collagen by western blot immediately after preparation (day 0), after 3 days of culturing (day 3) and after 3 days of treatment with C9 (day 3+C9). Bottom panel: densitometric quantification of procollagen. A.U., arbitrary densitometric units. B and C: analysis of additional independent preparations of slices.

Supplemental figure 5. Determination of α-smooth muscle actin (αSMA) in liver slices. A. Representative western blot of 4 untreated slices and 4 slices treated with C9. BHMT; betaine-homocysteine S-methyl transferase as loading control. B. Expression of αSMA normalized to expression of BHMT in 16 untreated and 16 C9 treated liver slices. Error bar +-1SD.

Supplemental figure 6. Effect of C9 in the prophylactic model; histology of additional livers (4x magnification). A. Sirius red staining of livers receiving vehicle. B. Sirius red staining of livers receiving C9. C. Sirius red staining of livers receiving C9 without fibrosis induction. D. Measurement of type I collagen and α-SMA by western blot. Liver samples were homogenized and total proteins were analyzed by western blot. Loading control: tubulin (TUB).

Supplemental figure 7. Effect of C9 in ethanol/$CCl_4$ therapeutic model; histology of additional livers (4x magnification). A. Sirius red staining of livers after 3 weeks of fibrosis induction. B. Sirius red staining of livers after 5 weeks of fibrosis induction and receiving vehicle in the final 2 weeks. C. Sirius red staining of livers after 5 weeks of fibrosis induction and receiving C9 in the final 2 weeks.

Supplemental figure 8. Effect of C9 in bile duct ligation prophylactic model; histology of

additional livers (4x magnification). A. Sirius red staining of livers after 2 days of bile duct ligation. B. Sirius red staining of livers after 10 days of bile duct ligation and receiving vehicle from day 2-10. C. Sirius red staining of livers after 10 days of bile duct ligation and receiving C9 from day 2-10.

Supplemental figure 9. Plasma concentration of C9 after oral gavage. 10 mg/kg of C9 was given by oral gavage in rats and plasma concentration of C9 was determined by mass spec after the indicated time points. N=3, error bars +-1SD.

SUP FIG 1

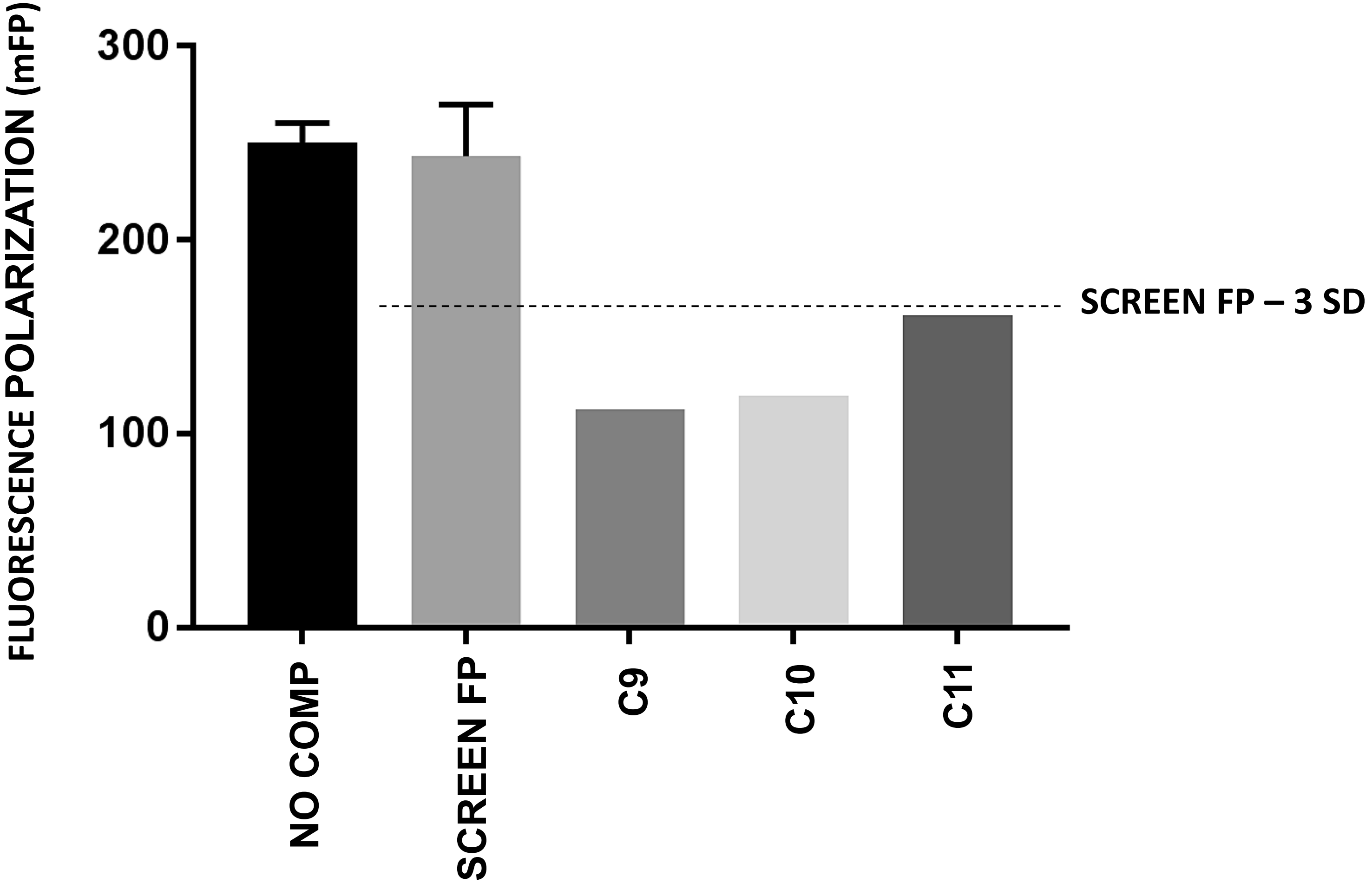
FLUORESCENCE POLARIZATION (mFP)
300
200
100
0
SCREEN FP – 3 SD
NO COMP
SCREEN FP
C9
C10
C11

SUP FIG 2

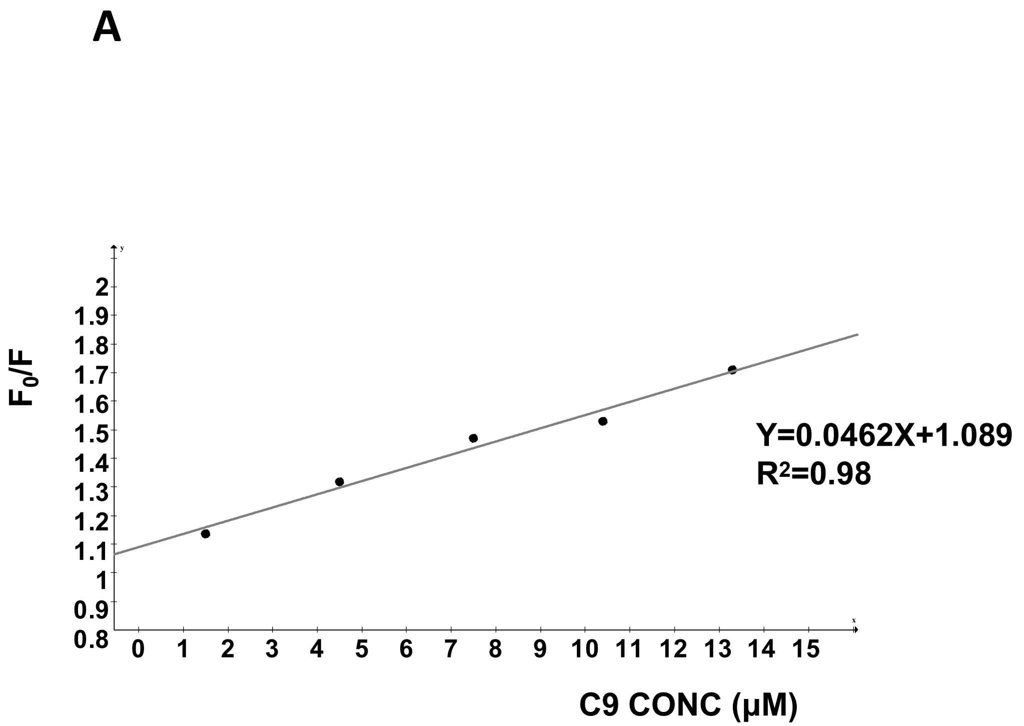

A
F0/F
2
1.9
1.8
1.7
1.6
1.5
1.4
1.3
1.2
1.1
1
0.9
0.8
0 1 2 3 4 5 6 7 8 9 10 11 12 13 14 15
C9 CONC (μM)
Y=0.0462X+1.089
R2=0.98


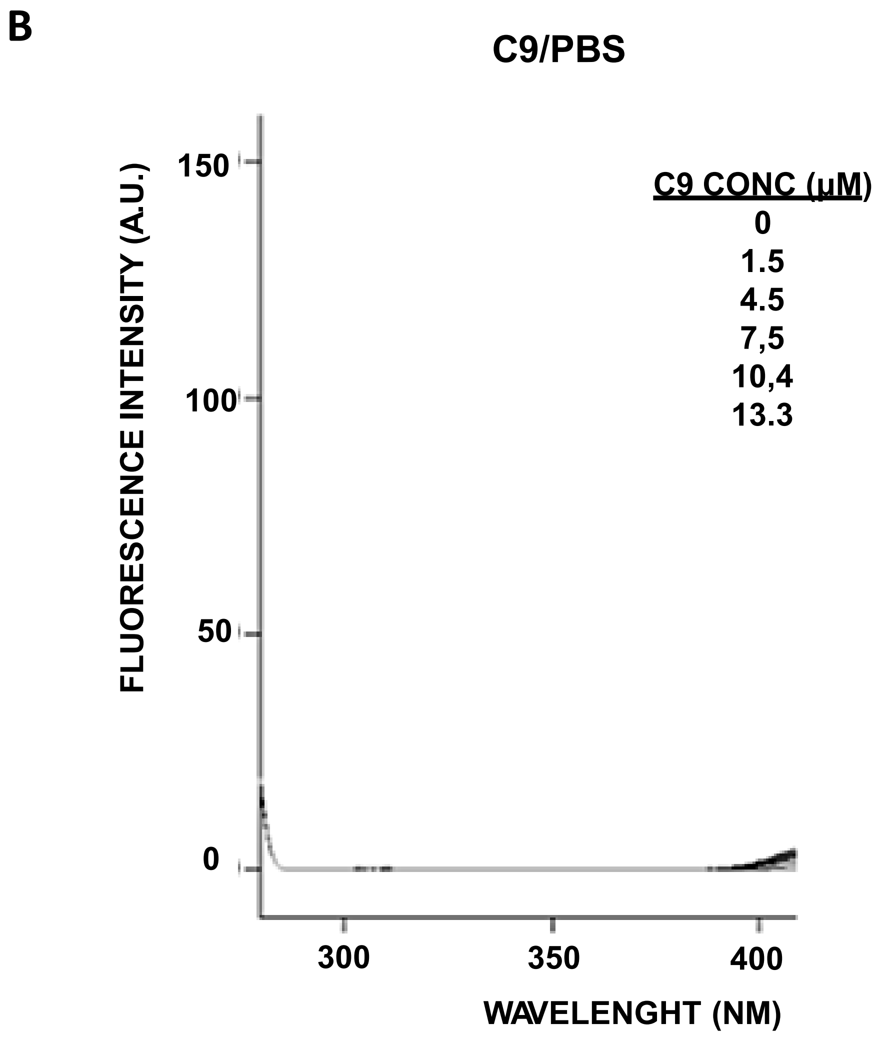

B
C9/PBS
FLUORESCENCE INTENSITY (A.U.)
150
100
50
0
300
350
400
WAVELENGHT (NM)
C9 CONC (μM)
0
1.5
4.5
7,5
10,4
13.3

SUP FIG 2

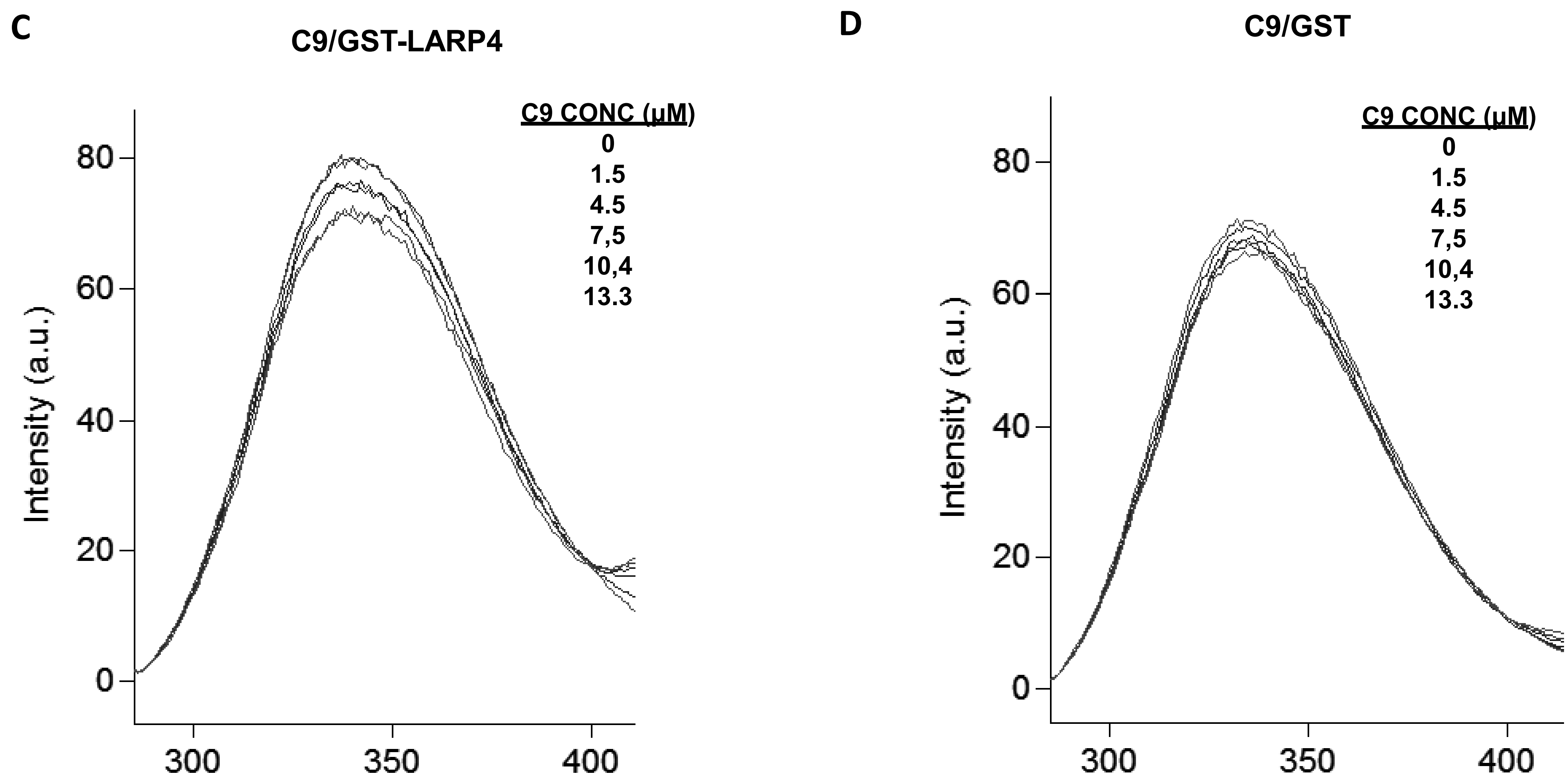
C
C9/GST-LARP4
C9 CONC (µM)
0
1.5
4.5
7,5
10,4
13.3
Intensity (a.u.)
80
60
40
20
0
300
350
400
D
C9/GST
C9 CONC (µM)
0
1.5
4.5
7,5
10,4
13.3
Intensity (a.u.)
80
60
40
20
0
300
350
400

SUP FIG 3

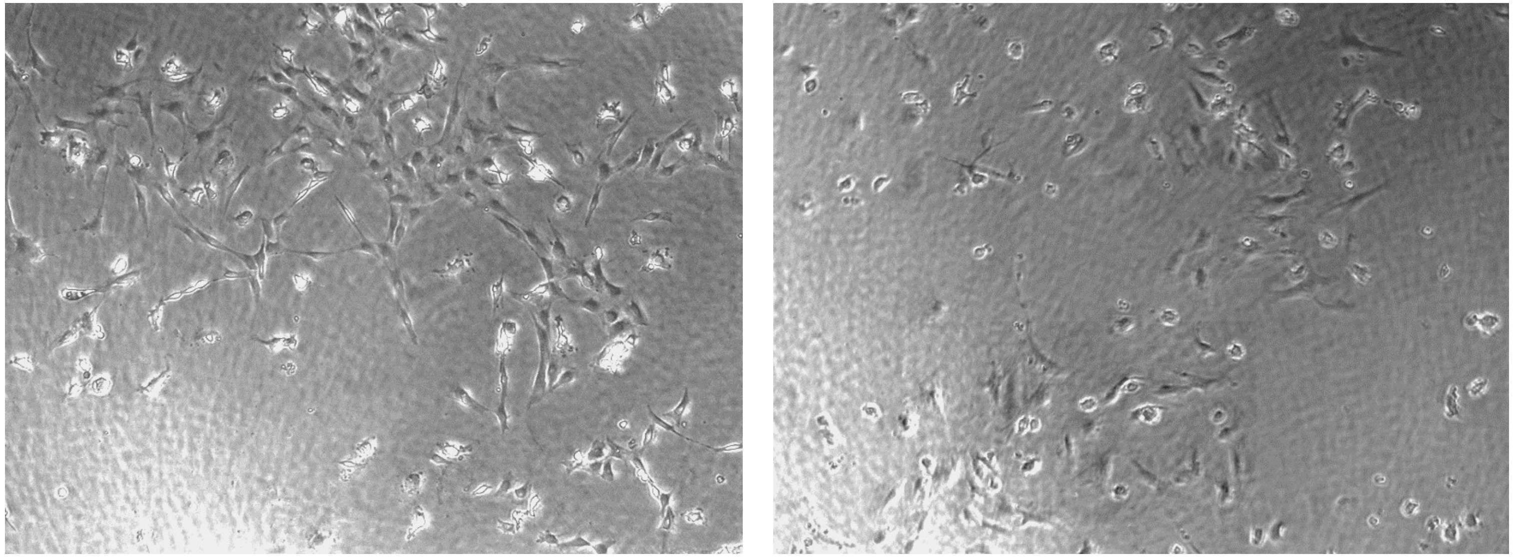

**Day 5 HSCs**

**Day 5 HSCs (100 nM C9)**

SUP FIG 4 A

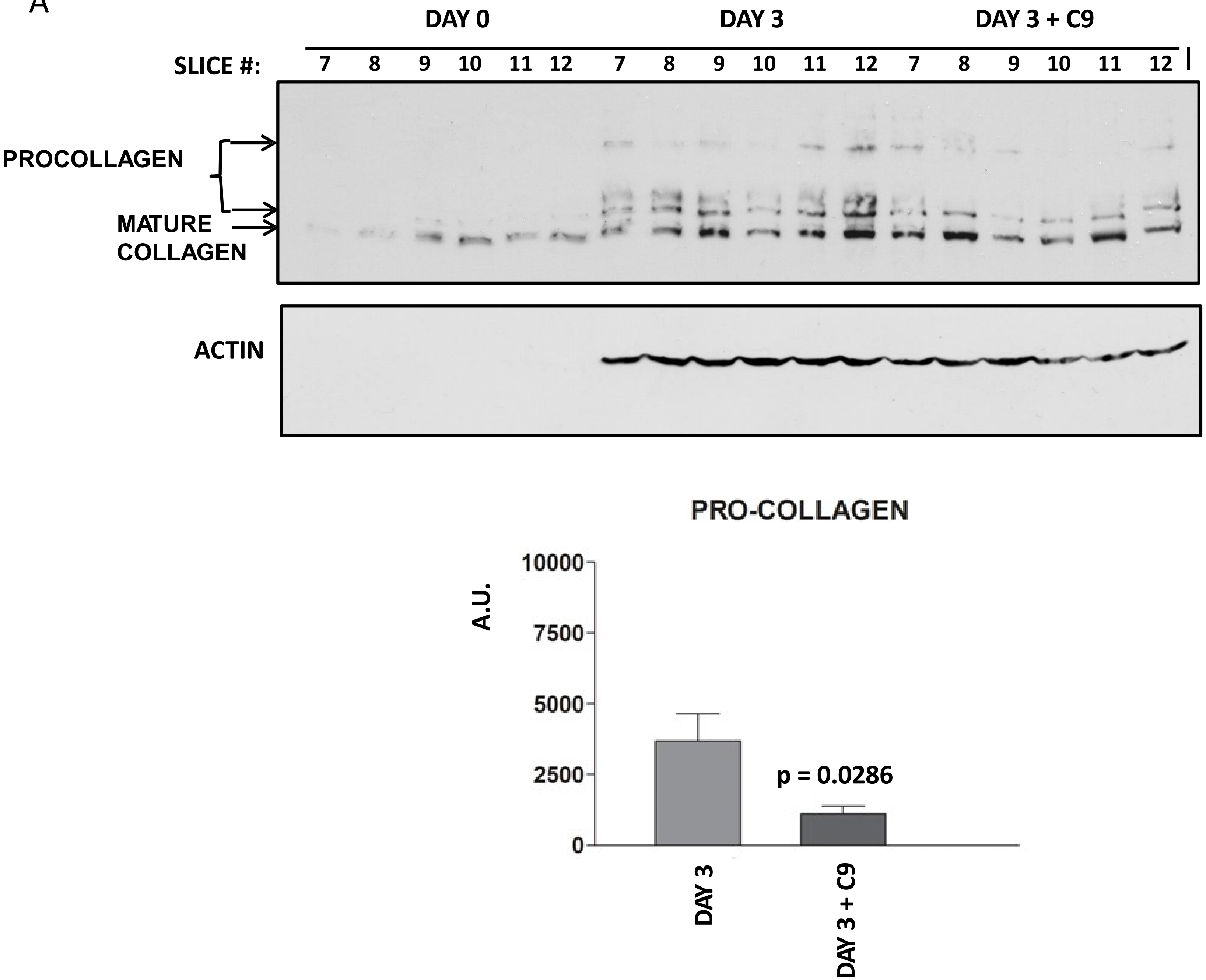
DAY 0
DAY 3
DAY 3 + C9
SLICE #:
7 8 9 10 11 12 7 8 9 10 11 12 7 8 9 10 11 12
PROCOLLAGEN
MATURE COLLAGEN
ACTIN
PRO-COLLAGEN
A.U.
10000
7500
5000
2500
0
p = 0.0286
DAY 3
DAY 3 + C9

SUP FIG 4 B

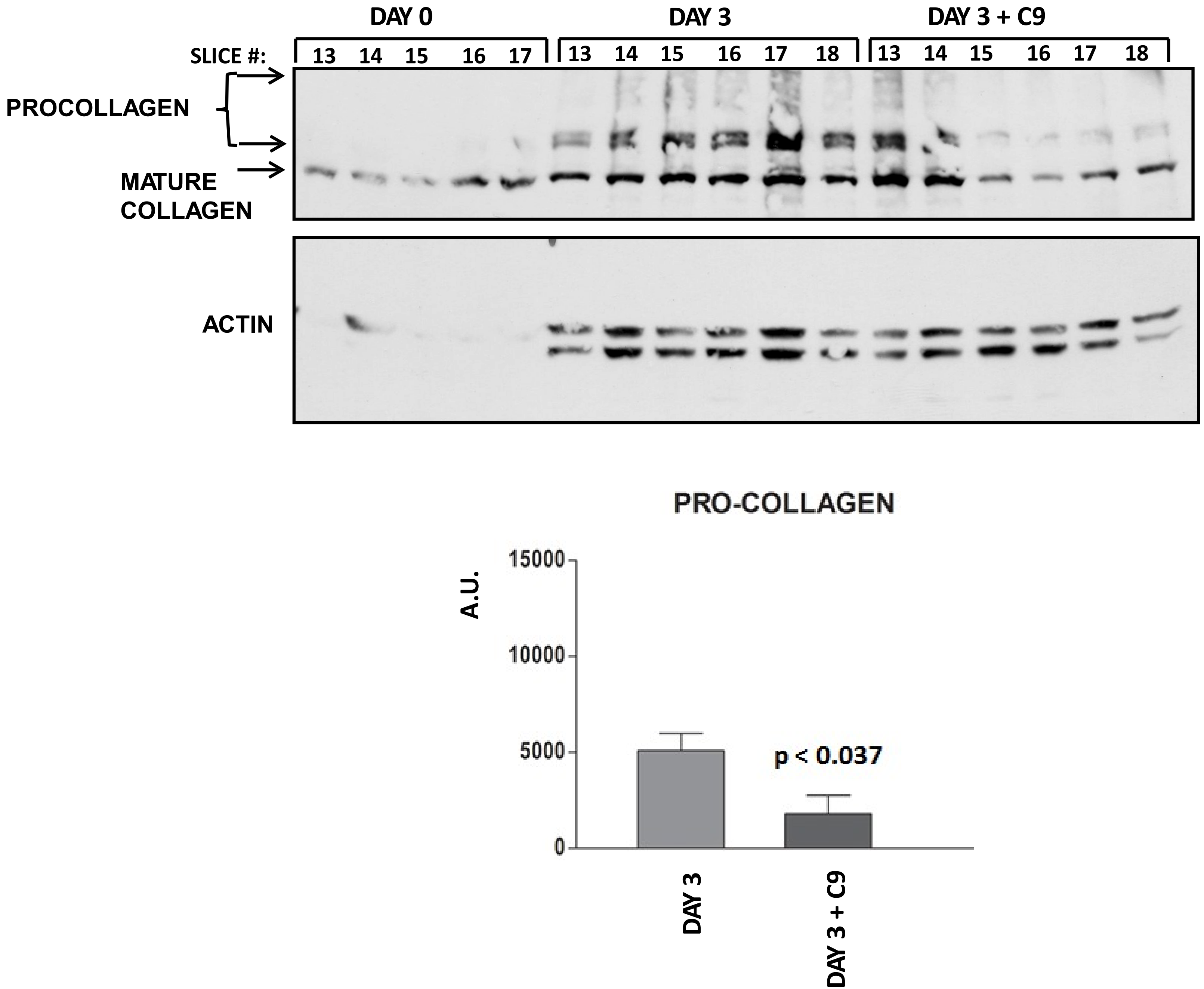
DAY 0
DAY 3
DAY 3 + C9
SLICE #:
13 14 15 16 17
13 14 15 16 17 18
13 14 15 16 17 18
PROCOLLAGEN
MATURE COLLAGEN
ACTIN
PRO-COLLAGEN
A.U.
15000
10000
5000
0
p < 0.037
DAY 3
DAY 3 + C9

SUP FIG 4

C

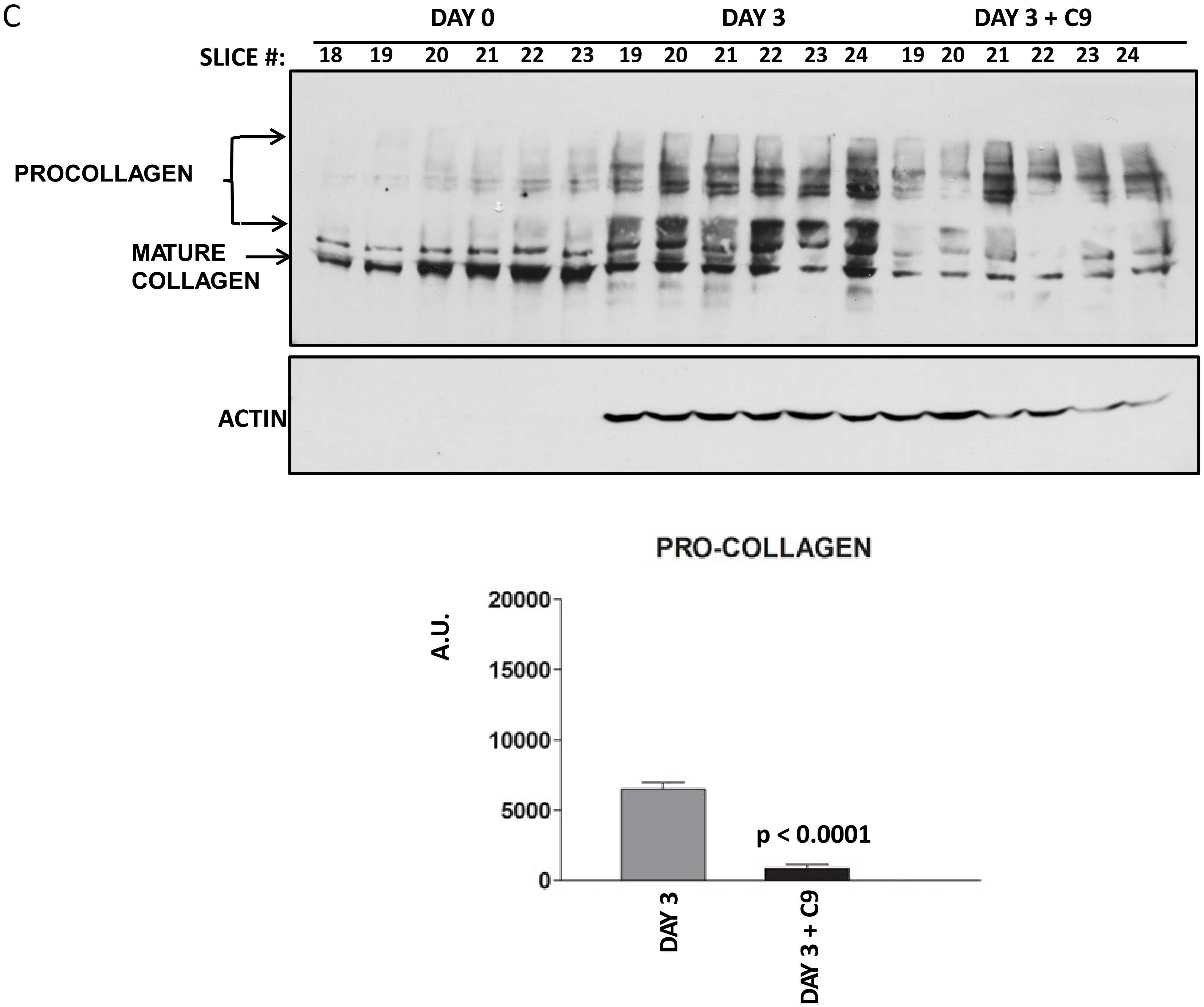
DAY 0
DAY 3
DAY 3 + C9
SLICE #:
18 19 20 21 22 23 19 20 21 22 23 24 19 20 21 22 23 24
PROCOLLAGEN
MATURE
COLLAGEN
ACTIN
PRO-COLLAGEN
A.U.
20000
15000
10000
5000
0
p < 0.0001
DAY 3
DAY 3 + C9

SUP FIG 5

A

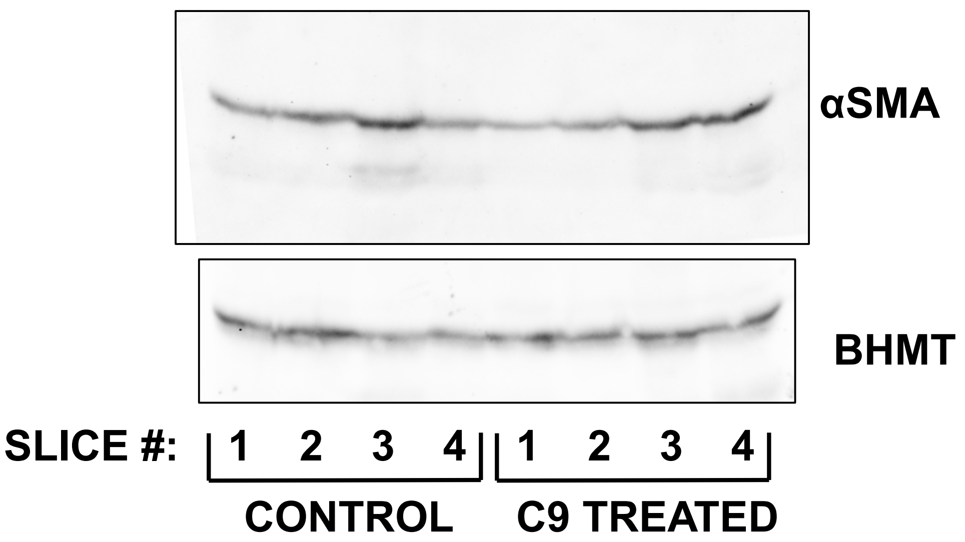
αSMA
BHMT
SLICE #:
1
2
3
4
1
2
3
4
CONTROL
C9 TREATED

B

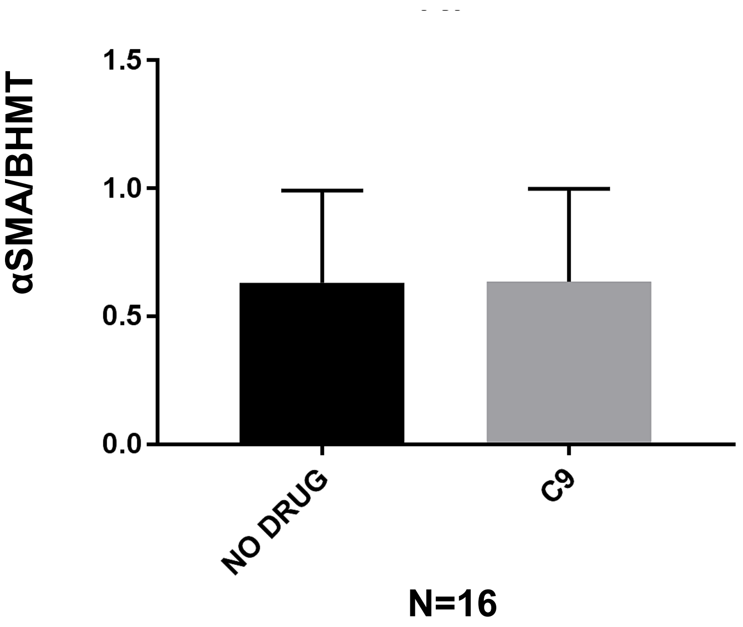
αSMA/BHMT
1.5
1.0
0.5
0.0
NO DRUG
C9
N=16

SUP FIG 6 A

4W ET/CCL4
+
4W VEH

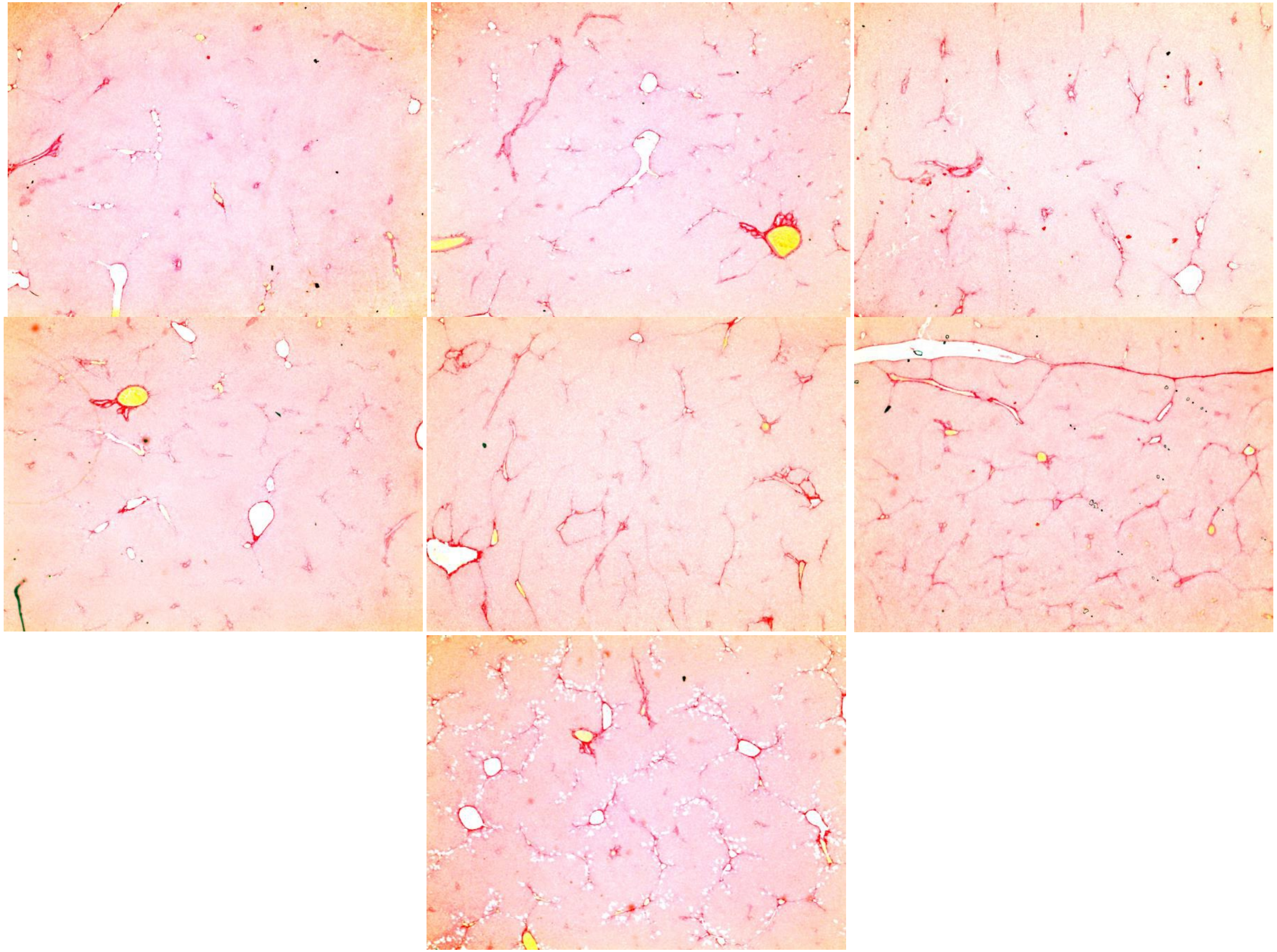

SUP FIG 6 B

**4W ET/CCL4**
**+**
**4W C9**

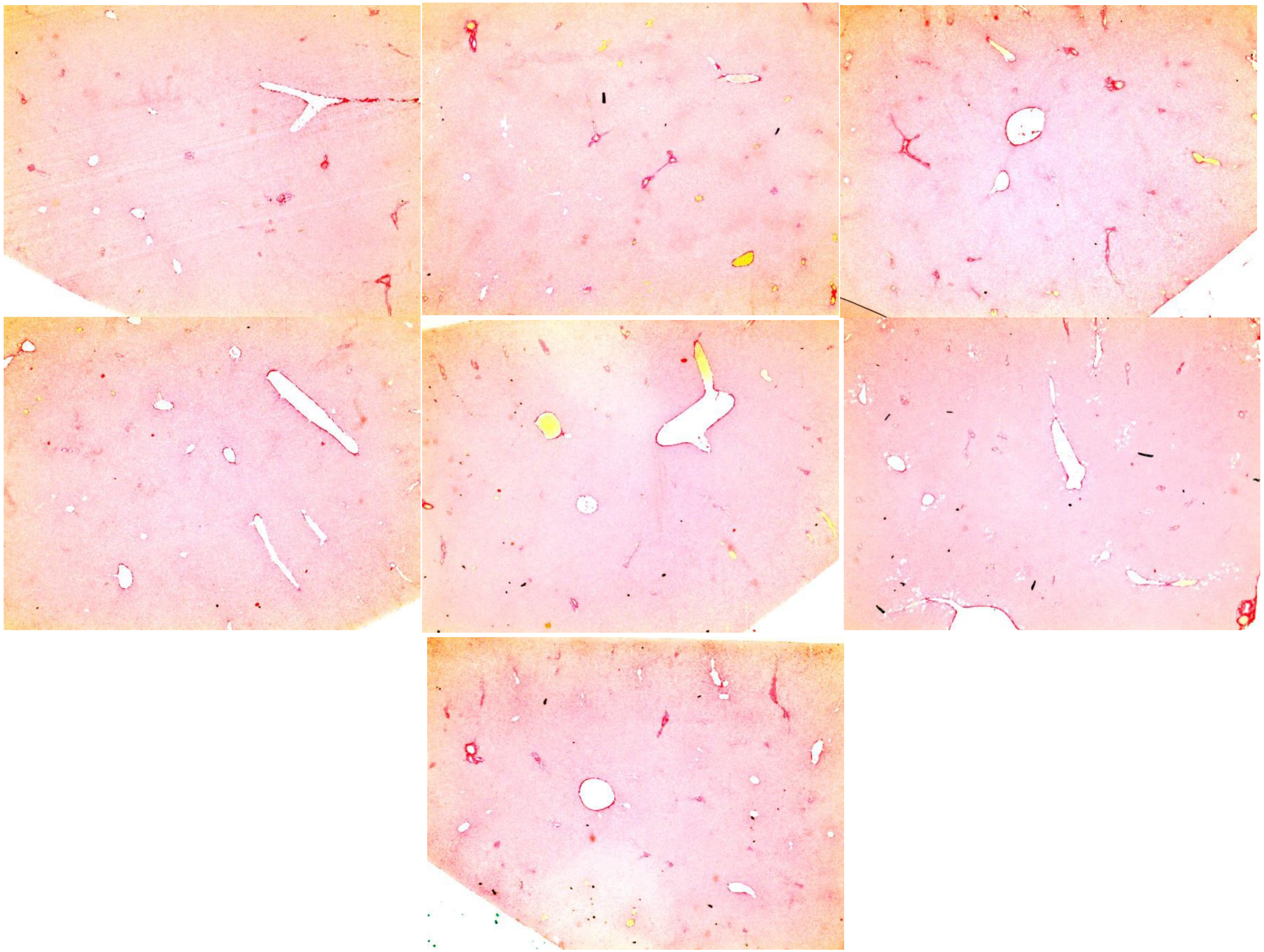

SUP FIG 6 C

**4W C9**

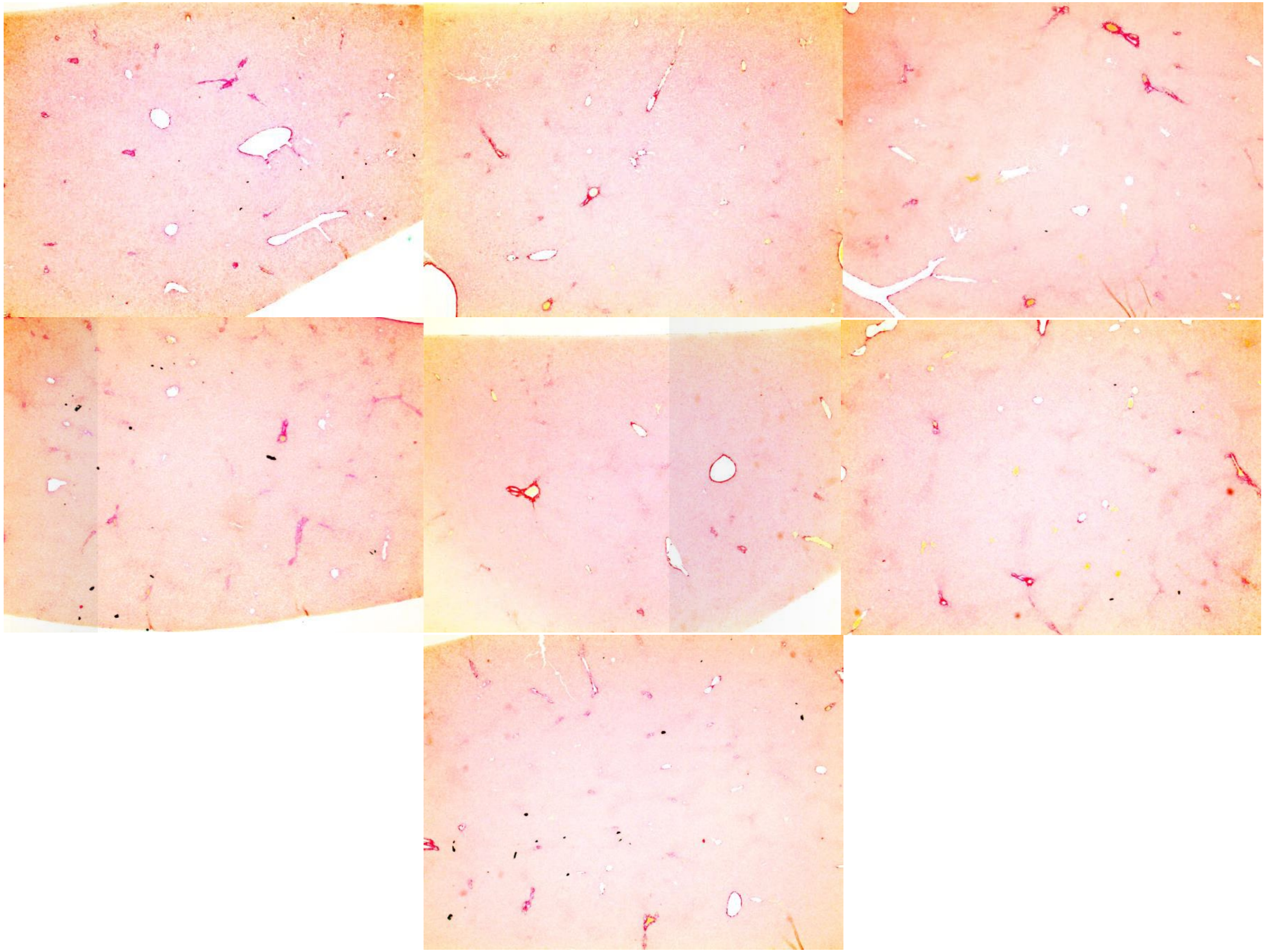

SUP FIG 6 D

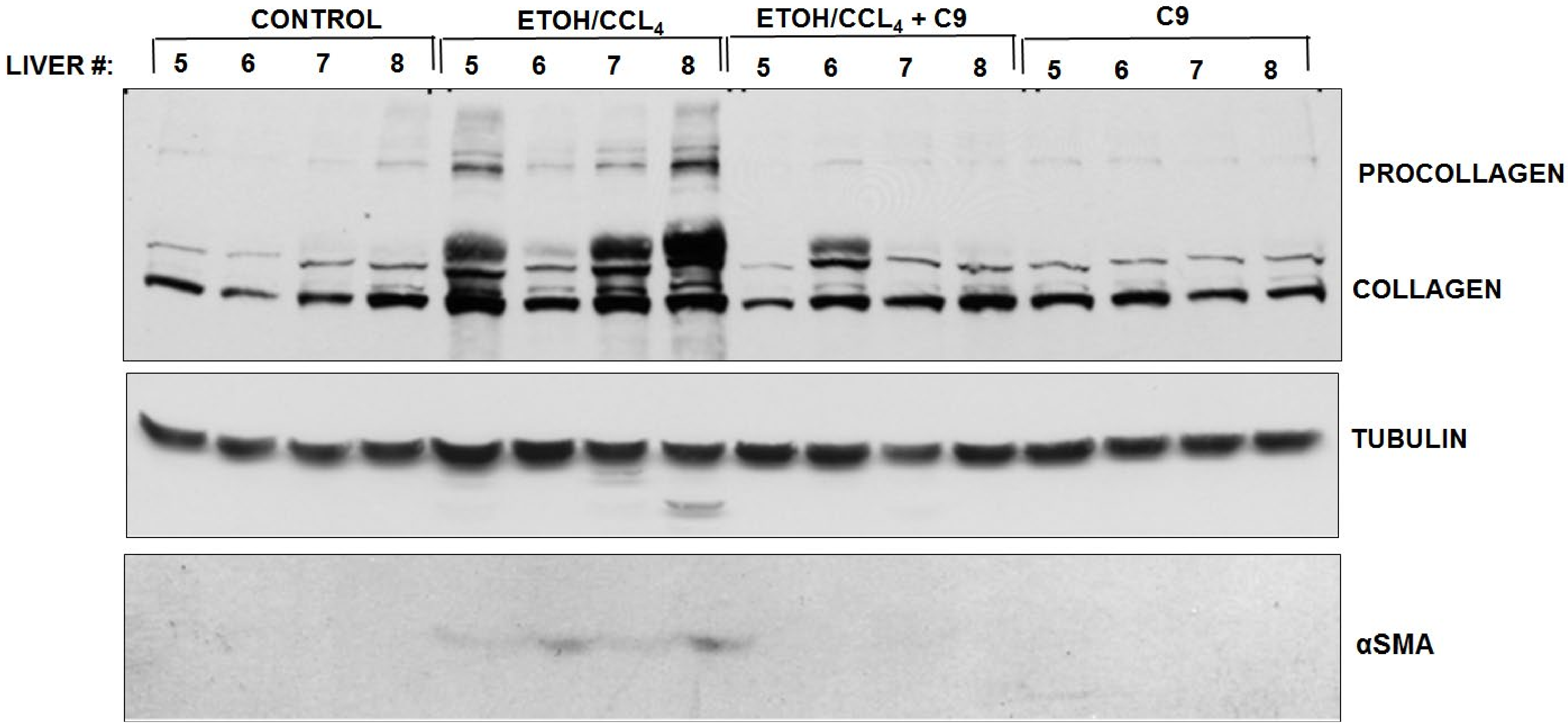
CONTROL
ETOH/CCL4
ETOH/CCL4 + C9
C9
LIVER #:
5 6 7 8 5 6 7 8 5 6 7 8 5 6 7 8
PROCOLLAGEN
COLLAGEN
TUBULIN
αSMA

SUP FIG 7 A

**3W ET/CCL4**

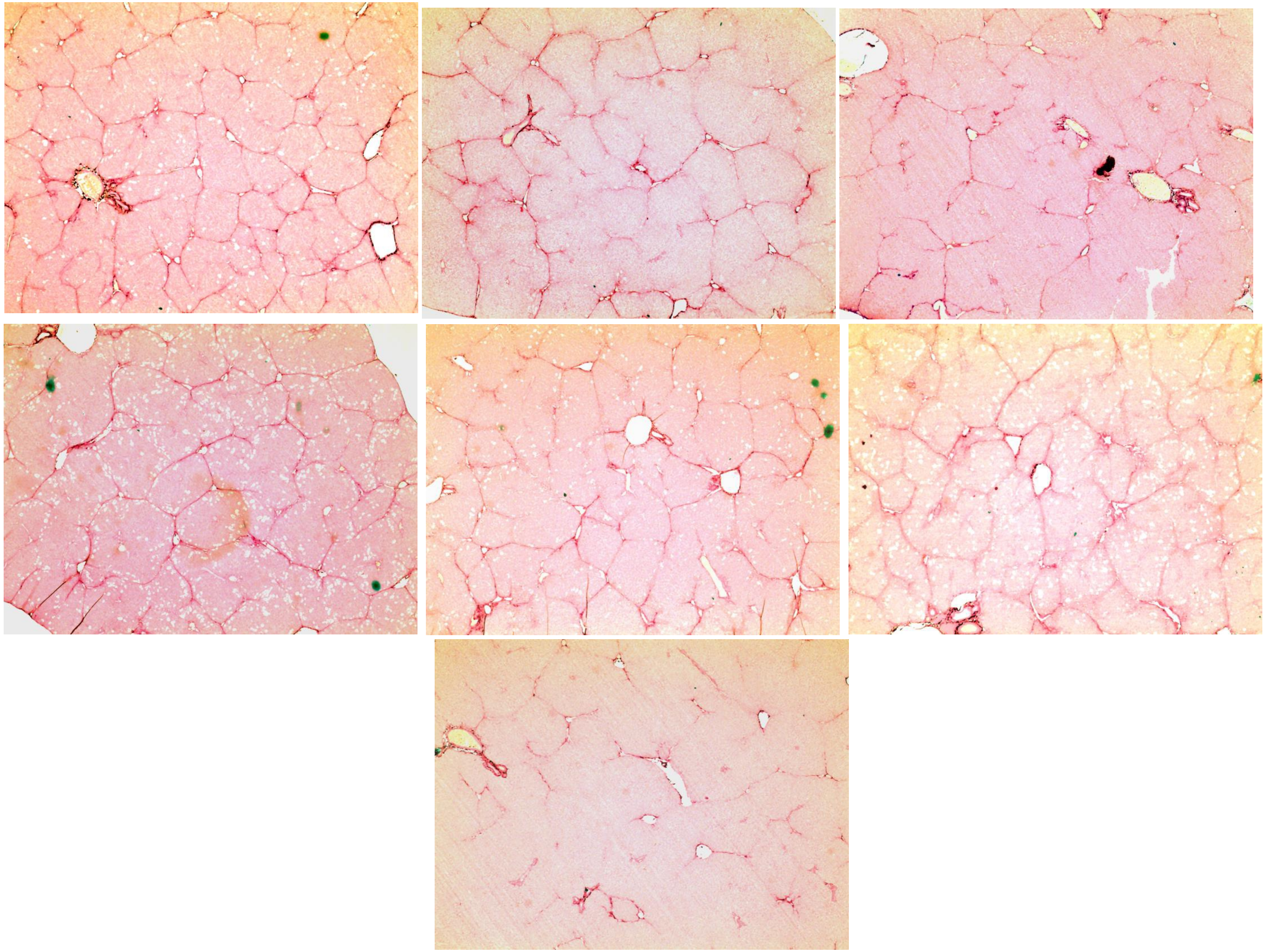

SUP FIG 7 B

**5W ET/CCL4**
**+**
**2W VEH**

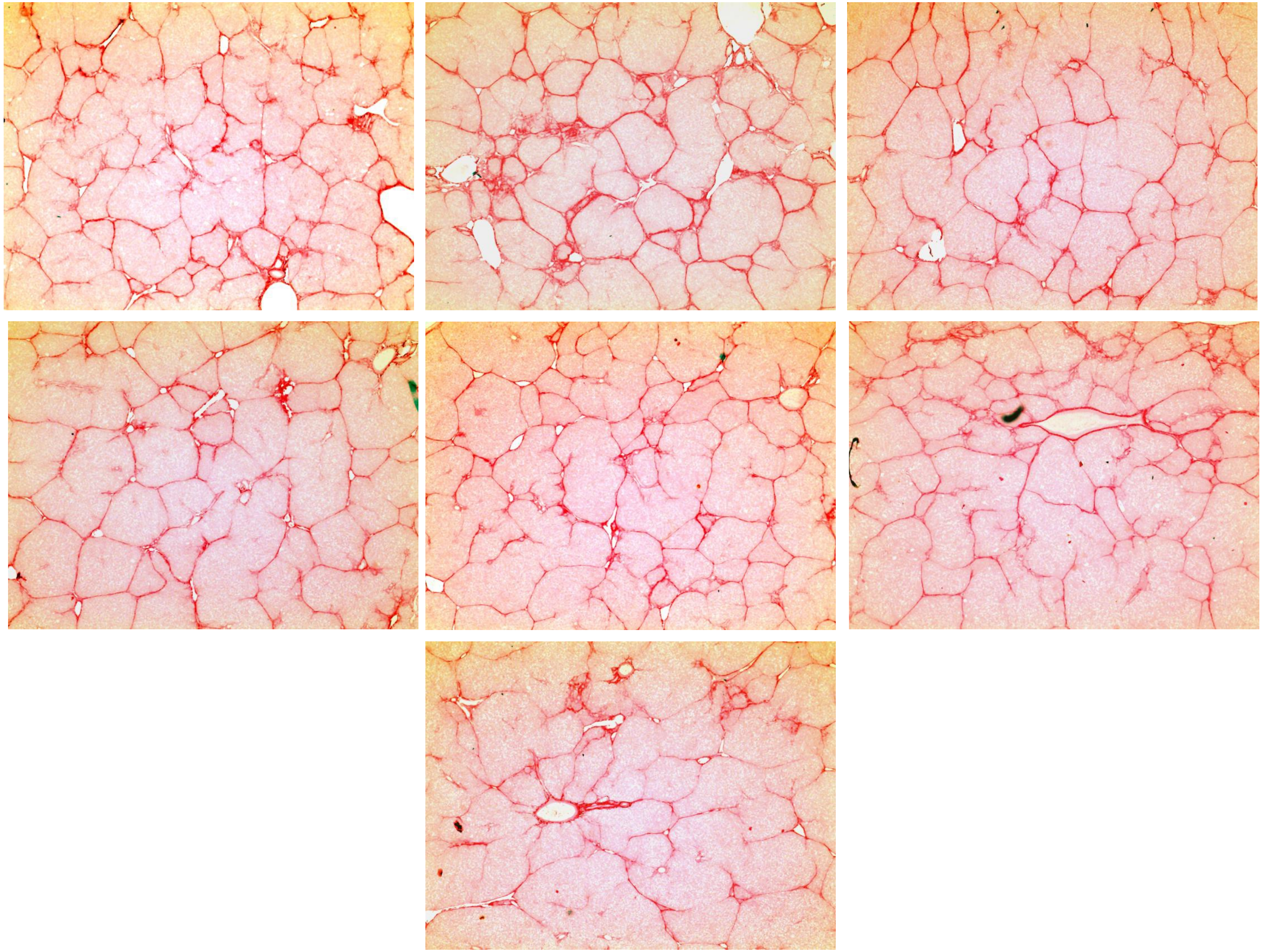

SUP FIG 7 C

**5W ET/CCL4**
**+**
**2W C9**

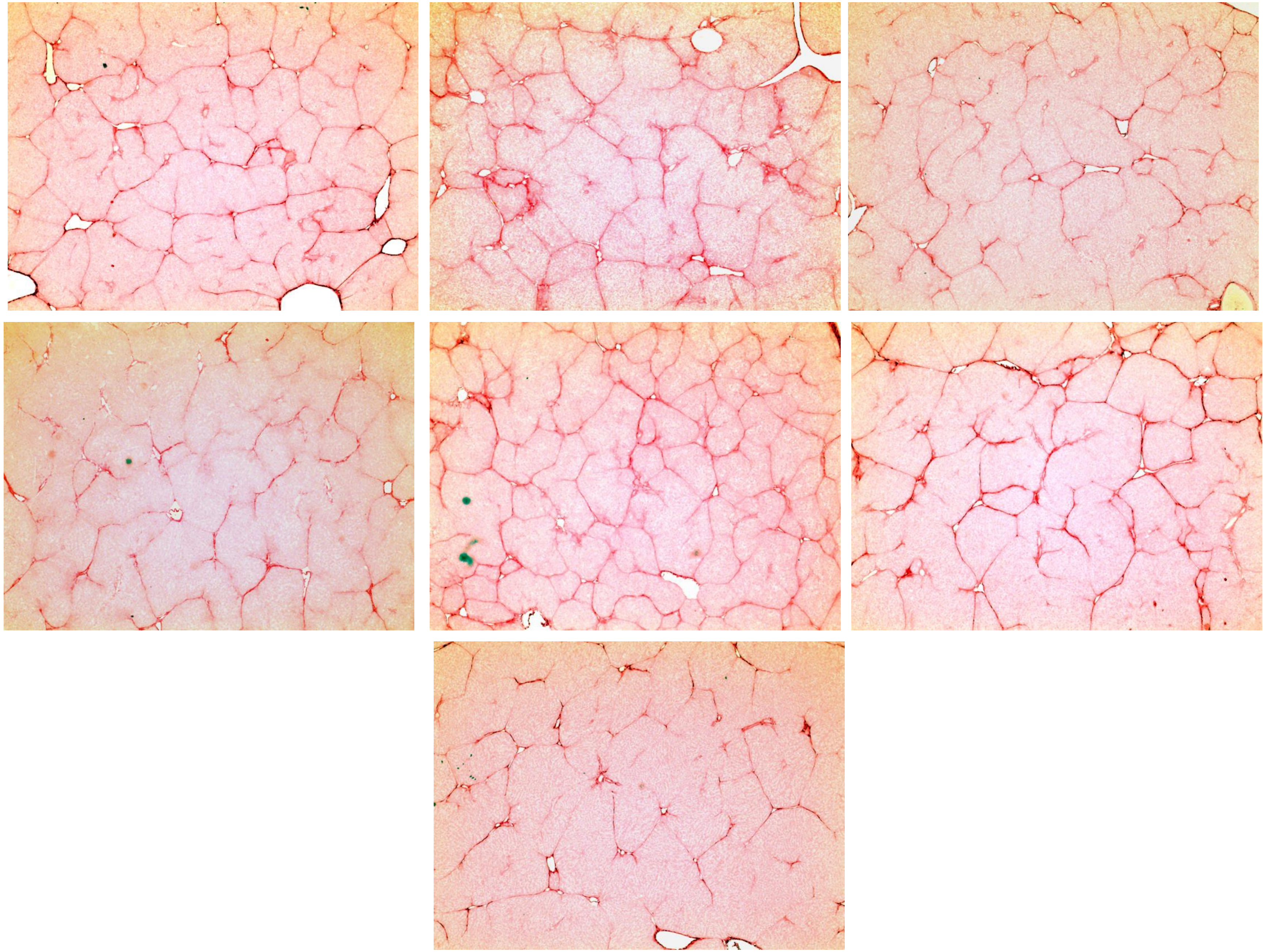

SUP FIG 8 A

**BDL DAY 2**

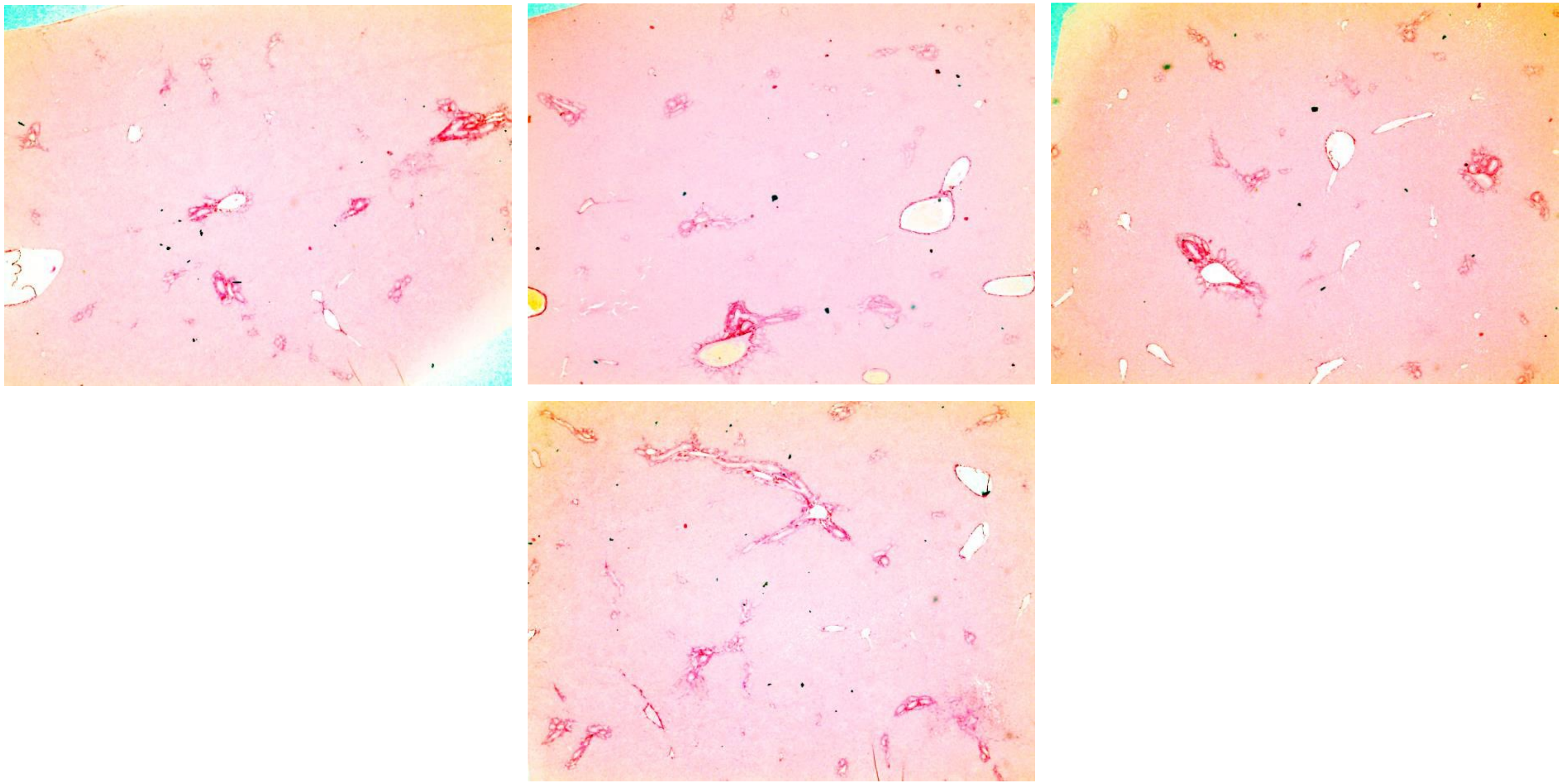

SUP FIG 8 B

**BDL DAY 10**
**+**
**VEH DAY 2-10**

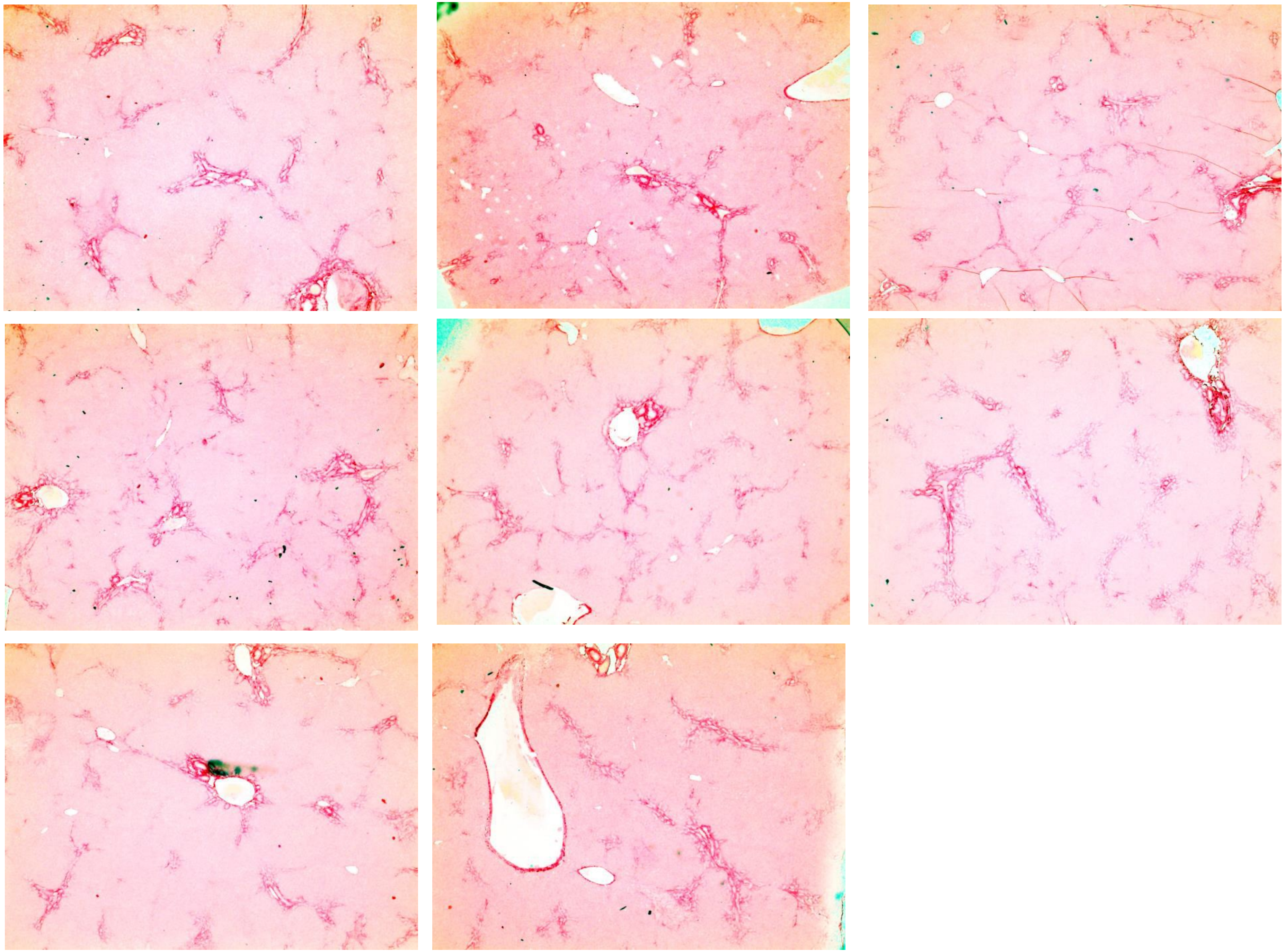

SUP FIG 8 C

**BDL DAY 10**
**+**
**C9 DAY 2-10**

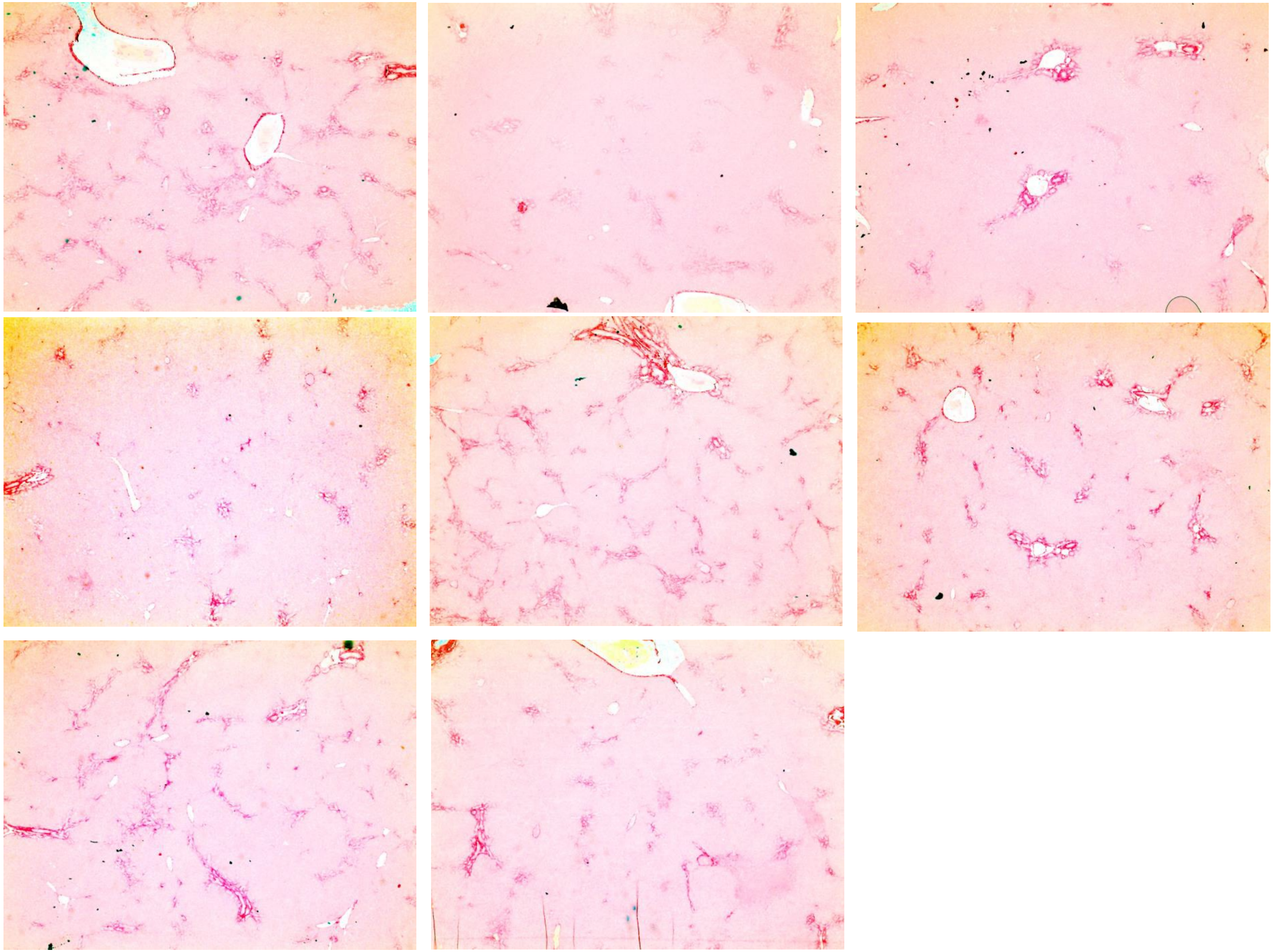

SUP FIG 9

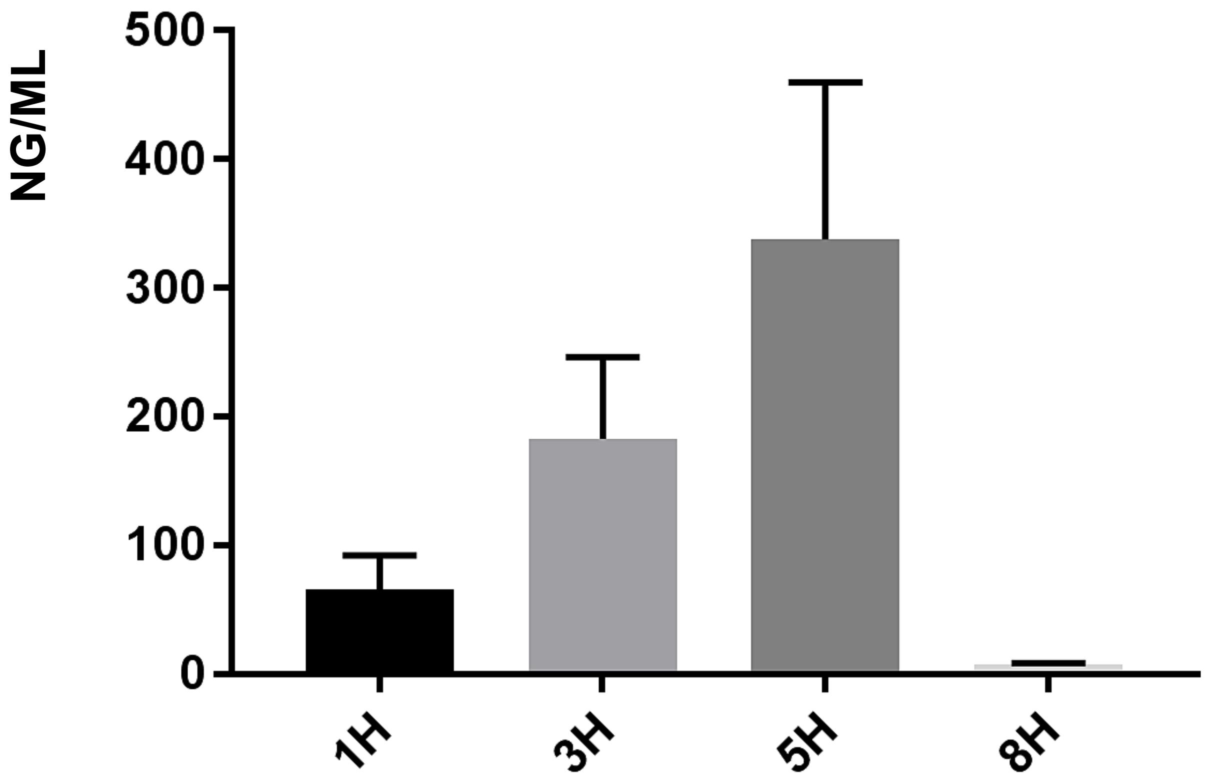

Supplemental Table 1. Changes in expression of liver genes after daily administration of C9 at 10 mg/kg for 4 weeks.

| Gene Symbol | Gene Name | p-value adj | Chane Dir. | Fold Change |
|---|---|---|---|---|
| Wfdc21 | WAP four-disulfide core domain 21 | 4.48E-10 | Up in Drug | 3.05 |
| Kng1l1 | kininogen 1-like 1 | 4.48E-10 | Up in Drug | 2.81 |
| Spink3 | serine peptidase inhibitor, Kazal type 3 | 1.80E-07 | Up in Drug | 2.75 |
| Cyp1a1 | cytochrome P450, family 1, subfamily a, polypeptide 1 | 1.80E-07 | Up in Drug | 2.74 |
| LOC100911558 | serine protease inhibitor Kazal-type 3-like | 1.05E-06 | Up in Drug | 2.39 |
| Adamdec1 | ADAM-like, decysin 1 | 1.90E-06 | Up in Drug | 2.54 |
| Kng2 | kininogen 2 | 2.37E-06 | Up in Drug | 2.27 |
| Spink1 | serine peptidase inhibitor, Kazal type 1 | 4.95E-05 | Up in Drug | 2.18 |
| Pla1a | phospholipase A1 member A | 0.000416376 | Up in Drug | 1.79 |
| Acsf2 | acyl-CoA synthetase family member 2 | 0.000416376 | Up in Drug | 1.53 |
| Pcolce | procollagen C-endopeptidase enhancer | 0.000416395 | Up in Drug | 2.04 |
| Trpm4 | transient receptor potential cation channel, subfamily M, member 4 | 0.00041659 | Up in Drug | 1.90 |
| Prph | peripherin | 0.000606699 | Up in Drug | 2.14 |
| Socs3 | suppressor of cytokine signaling 3 | 0.00101044 | Up in Drug | 1.96 |
| Ethe1 | ethylmalonic encephalopathy 1 | 0.001138609 | Up in Drug | 1.67 |
| Il7 | interleukin 7 | 0.002072783 | Up in Drug | 1.96 |
| Cd36 | CD36 molecule (thrombospondin receptor) | 0.004196454 | Up in Drug | 1.83 |
| Bhmt | betaine-homocysteine S-methyltransferase | 0.004196454 | Up in Drug | 1.63 |
| Ccdc64 | coiled-coil domain containing 64 | 0.009457919 | Up in Drug | 1.79 |
| Ddhd1 | DDHD domain containing 1 | 0.014814389 | Up in Drug | 1.87 |
| RGD1565355 | similar to fatty acid translocase/CD36 | 0.020758642 | Up in Drug | 1.75 |
| Lbp | lipopolysaccharide binding protein | 0.020758642 | Up in Drug | 1.41 |
| Ormdl1 | ORMDL sphingolipid biosynthesis regulator 1 | 0.022665537 | Up in Drug | 1.58 |
| Pla2g4b | phospholipase A2, group IVB (cytosolic) | 0.022665537 | Up in Drug | 1.54 |
| Unc5cl | unc-5 homolog C (C. elegans)-like | 0.029611753 | Up in Drug | 1.80 |
| Enpp5 | ectonucleotide pyrophosphatase/phosphodiesterase 5 | 0.029611753 | Up in Drug | 1.47 |
| Tnfrsf9 | tumor necrosis factor receptor superfamily, member 9 | 0.029882766 | Up in Drug | 1.79 |
| Orm1 | orosomucoid 1 | 0.029882766 | Up in Drug | 1.78 |
| Sstr3 | somatostatin receptor 3 | 0.032007021 | Up in Drug | 1.69 |
| Sema4a | sema domain, immunoglobulin domain (Ig), transmembrane domain (TM | 0.04922708 | Up in Drug | 1.55 |
| Prg4 | proteoglycan 4 | 0.04922708 | Up in Drug | 1.41 |

| Gene Symbol | Gene Name | p-value adj | Chane Dir. | Fold Change |
|---|---|---|---|---|
| Ptma* | prothymosin alpha | 5.78E-05 | Down in Drug | 1.60 |
| Cds1 | CDP-diacylglycerol synthase 1 | 0.002072783 | Down in Drug | 1.66 |
| Rhob | ras homolog family member B | 0.00338812 | Down in Drug | 1.65 |
| Ptma* | prothymosin alpha | 0.003419803 | Down in Drug | 1.69 |
| Gstm3 | glutathione S-transferase mu 3 | 0.004437057 | Down in Drug | 1.95 |
| Ar | androgen receptor | 0.008235537 | Down in Drug | 1.87 |
| Prmt2 | protein arginine methyltransferase 2 | 0.009457919 | Down in Drug | 1.60 |
| Nim1k | NIM1 serine/threonine protein kinase | 0.022665537 | Down in Drug | 1.68 |
| Copg1 | coatomer protein complex, subunit gamma 1 | 0.032007021 | Down in Drug | 1.42 |
| Abcb1b | ATP-binding cassette, subfamily B (MDR/TAP), member 1B | 0.034343998 | Down in Drug | 1.80 |
| Txndc5 | thioredoxin domain containing 5 (endoplasmic reticulum) | 0.04922708 | Down in Drug | 1.48 |
| | | | | |
| | | | | |

*Ptma is listed as the gene name for two Ensembl gene IDs: ENSRNOG00000018584 and ENSRNOG00000025731. Unique reads mapped to each ID.
